# Optimize Before You Synthesize—Enhancing the Ionic Conductivity of Li_7_SiPS_8_ Using Bayesian Optimization

**DOI:** 10.1002/anie.5778118

**Published:** 2026-05-23

**Authors:** Lucas G. Balzat, Robert Calaminus, Yinghan Zhao, Kristina Gjorgjevikj, Igor Moudrakovski, Simon Krause, Arnd Koeppe, Britta Nestler, Bettina V. Lotsch

**Affiliations:** ^1^ Department of Nanochemistry Max Planck Institute for Solid State Research Stuttgart Germany; ^2^ Department of Chemistry Ludwig Maximilian University Munich Munich Germany; ^3^ Institute for Applied Materials ‐ Microstructure Modelling and Simulation Karlsruhe Institute of Technology Karlsruhe Germany; ^4^ Amazon Development Center Germany GmbH Berlin Germany; ^5^ Institute for Inorganic Chemistry II Ulm University Ulm Germany

**Keywords:** bayesian optimization, electrochemistry, ionic conductivity, solid electrolyte, synthesis design

## Abstract

Tetragonal ${\mathrm{Li}}_{7}{\mathrm{SiPS}}_{8}$ is a superionic solid electrolyte, yet its Li ion conductivity suffers from the presence of an amorphous side phase. Attempts to optimize the ionic conductivity, however, are incremental and hence time‐consuming, because the relationship between synthesis conditions and electrolyte performance is largely unknown. In this work, we employ Bayesian optimization (BO) as an efficient design‐of‐experiment approach to increase the ionic conductivity of the ${\mathrm{Li}}_{7}{\mathrm{SiPS}}_{8}$ system. Our data‐driven workflow reproducibly yields ${\mathrm{Li}}_{7}{\mathrm{SiPS}}_{8}$ with ionic conductivities exceeding 7 mS ${\mathrm{cm}}^{-1}$ at room temperature, an increase by up to 350% compared to previously reported routes. Simultaneously, the optimized solid‐state synthesis lowered the synthesis temperature by 100 K (20%) and shortened the reaction time by 76 h (76%), delivering a more energy‐efficient and, hence, sustainable process. To probe the origin of the increased conductivity, we examined six representative samples by quantitative Rietveld refinements, synchrotron x‐ray powder diffraction, pair distribution function analysis, solid‐state and pulsed‐field‐gradient NMR, electron microscopy, and Raman spectroscopy. We demonstrate that BO can help navigate the complex synthesis parameter space, thereby accelerating the development of high‐performance sulfide electrolytes for next‐generation batteries.

## Graphical abstract

Bayesian optimization was used to optimize the synthesis parameters of the solid electrolyte ${\mathrm{Li}}_{7}{\mathrm{SiPS}}_{8}$ in order to increase the ionic conductivity. After only 32 iterations, the ionic conductivity was successfully increased from 2 to 7 mS ${\mathrm{cm}}^{-1}$ at 25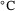, while synthesis temperature and time were both significantly reduced.

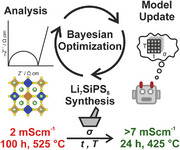

## 1 Introduction

Solid‐state batteries (SSBs) have been hailed as a key technology for more sustainable, more efficient, and better‐performing energy storage systems. Compared to traditional Li‐ion batteries (LIBs), SSBs promise higher energy densities, improved safety, and longer cycle stability [1, 2, 3]. The essential component of SSBs is the solid electrolytes (SE), some of which show ionic conductivities comparable to those of with liquid electrolytes commonly used in conventional LIBs [1, 2, 4, 5, 6, 7]. However, in order to enable fast charging (high C rates), while also maintaining reasonable energy densities, SEs with an ionic conductivity of 10 mS ${\mathrm{cm}}^{-1}$ are required [3, 6, 8]. Hence, maximizing overall ionic conductivity is the main goal.

Currently, most SEs breaching the 10 mS ${\mathrm{cm}}^{-1}$ threshold belong to the class of thiophosphates, like halide‐rich argyrodites (${\mathrm{Li}}_{5.5}{\mathrm{PS}}_{4.5}{\mathrm{Cl}}_{1.5}$) [9] or LGPS (${\mathrm{Li}}_{10}{\mathrm{GeP}}_{2}S_{12}$) and other structurally related compounds [7, 10, 11, 12, 13]. One such LGPS‐related compound is tetragonal ${\mathrm{Li}}_{7}{\mathrm{SiPS}}_{8}$ (*tetra*‐${\mathrm{Li}}_{7}{\mathrm{SiPS}}_{8}$), in which the expensive and scarce element germanium is replaced with low‐cost and earth‐abundant silicon [14, 15, 16]. The crystal structure of *tetra*‐${\mathrm{Li}}_{7}{\mathrm{SiPS}}_{8}$ features a characteristic arrangement of ${\mathrm{SiS}}_{4}$ and ${\mathrm{PS}}_{4}$ tetrahedra and can be understood as an ordered variant of a solid solution between ${\mathrm{Li}}_{4}{\mathrm{SiS}}_{4}$ [17, 18, 19] and β‐${\mathrm{Li}}_{3}{\mathrm{PS}}_{4}$. Here, the ${\mathrm{PS}}_{4}$ and ${\mathrm{SiS}}_{4}$ tetrahedra are not statistically distributed as they are in the orthorhombic polymorph (*ortho‐* ${\mathrm{Li}}_{7}{\mathrm{SiPS}}_{8}$) [14, 20, 21]. However, the ionic conductivity of *tetra*‐${\mathrm{Li}}_{7}{\mathrm{SiPS}}_{8}$ derived from electrochemical impedance spectroscopy (EIS) is reported to be “only” 2 mS ${\mathrm{cm}}^{-1}$ [14]. In contrast, predictions by Ong et al. and pulsed field gradient (PFG) NMR experiments by Harm et al. suggest an ionic conductivity for *tetra*‐${\mathrm{Li}}_{7}{\mathrm{SiPS}}_{8}$ of 5±1 $\mathrm{mS}\;{\mathrm{cm}}^{-1}$ [14, 22]. Harm et al. attributed this discrepancy in conductivity to the presence of a poorly ion‐conducting, amorphous side phase [14]. Amorphous side phases are known to affect ionic conductivities of SEs, but Harm et al. could not draw a definitive conclusion regarding the influence of the side phase on the ionic conductivity of *tetra*‐${\mathrm{Li}}_{7}{\mathrm{SiPS}}_{8}$ [12, 14, 23, 24].

This difference between the predicted and reported ionic conductivities prompted us to investigate whether the ionic conductivity of *tetra*‐${\mathrm{Li}}_{7}{\mathrm{SiPS}}_{8}$ could be improved. However, conventional trial‐and‐error approaches are often slow, costly, and inefficient in addressing complex parameter spaces [25]. To overcome these challenges, machine learning (ML) and artificial intelligence (AI) have emerged as powerful tools for accelerating materials discovery and optimization [26, 27, 28]. In particular, Bayesian optimization (BO) provides an effective framework for navigating complex, expensive to‐evaluate “black‐box” functions, which are frequently encountered in materials science [29]. This approach enables rapid optimization of material properties and processing parameters. BO is particularly well‐suited for the discovery and optimization of solid‐state battery materials, where the relationships between input parameters (e.g., composition, temperature, and synthesis time) and output properties (e.g., ionic conductivity and stability) are often highly nonlinear, poorly understood, and expensive to evaluate experimentally [30]. Hence, BO can be used to efficiently improve synthesis conditions of SEs in order to increase a target value, that is, the ionic conductivity. While such approaches have been reported for oxide‐based SEs, only a few cases addressing sulfide‐based SEs are known [31, 32, 33, 34].

In this study, we employed BO to efficiently identify optimal synthesis conditions (temperature and time) that maximize the ionic conductivity of *tetra*‐${\mathrm{Li}}_{7}{\mathrm{SiPS}}_{8}$, while a surrogate model was iteratively updated using all previously collected experimental data to guide the search for improved synthesis parameters. By leveraging BO, we reduced the synthesis temperature and time of *tetra*‐${\mathrm{Li}}_{7}{\mathrm{SiPS}}_{8}$ from 798 K and 100 h to 698 K and 24 h, respectively, resulting in a more energy‐ and time‐efficient synthesis. Furthermore, the optimized synthesis conditions resulted in a reproducible increase of the ionic conductivity from 2 to over 7 mS ${\mathrm{cm}}^{-1}$. Since BO does not impose an explicit mechanistic model on the underlying structure–property relationships, but rather models the target property probabilistically, we selected six representative samples to further gain insight into the mechanism behind the conductivity increase. The six representative samples included both fast‐ and slow‐conducting ${\mathrm{Li}}_{7}{\mathrm{SiPS}}_{8}$ samples, which were analyzed by (synchrotron‐) powder x‐ray diffraction (PXRD), pair distribution function (PDF) analysis, solid‐state and PFG‐NMR, scanning electron microscopy (SEM)/energy‐dispersive x‐ray spectroscopy (EDX), and Raman spectroscopy. Interestingly, the measured structural and spectroscopic data, as well as the ionic conductivity, showed no clear systematic correlation. This apparent absence of a clear structure–property correlation highlights the underlying complexity of ion conduction in sulfide solid electrolytes and underscores the value of Bayesian‐based approaches for optimizing performance in systems where complex interrelations between multiple parameters render mechanistic insights challenging.

## 2 Results and Discussion

### 2.1 Bayesian Optimization of the Ionic Conductivity of ${\mathrm{Li}}_{7}{\mathrm{SiPS}}_{8}$

The optimization of ionic conductivity as a function of synthesis temperature and time was performed using a BO framework. BO was implemented using a Gaussian process (GP) surrogate model with a Matérn 5/2 kernel and an expected improvement acquisition function to balance exploration and exploitation (see Supporting Information for details). The process was initialized using two experimental procedures for synthesizing *tetra*‐${\mathrm{Li}}_{7}{\mathrm{SiPS}}_{8}$ as reported in earlier studies [14, 35]. In this study, we randomly selected 10 synthesis conditions from a range of experimental conditions of interest to synthesize initial ${\mathrm{Li}}_{7}{\mathrm{SiPS}}_{8}$ samples. This was followed by the determination of amorphous and crystalline phase composition *via* Rietveld analysis employing an internal silicon standard (see Figures S1–S11, and Tables S2–S12) and ionic conductivity using EIS. All EIS data are shown in the Supporting Information (Figures S12–S18). In each subsequent iteration, the GP surrogate model was updated with conductivity results from the last iteration. In each iteration, two new synthesis conditions based on the acquisition function were sequentially proposed, with the first suggested point temporarily added to the dataset with an artificial placeholder result to avoid duplicate sampling, enabling parallel experimentation. Over the course of optimization, a total of 32 experiments were conducted, as summarized in Table S1. While it is common to revisit each data point several times to obtain more reliable conductivities, it was not possible to synthesize samples multiple times due to time and resource constraints. Therefore, only the synthesis conditions yielding the highest conductivities were revisited and reproduced [36]. More information regarding the BO process is given in the supporting information. The BO‐guided search rapidly converged on a high‐performance synthesis window. The highest ionic conductivity achieved was $7.43\left(\pm 0.50\%\right)\;\mathrm{mS}\;{\mathrm{cm}}^{-1}$ for a sample synthesized at 698 K for 24 h (Index 32). This result was consistent with other experiments in the same parameter region, including 7.25(±0.33%) $\mathrm{mS}\;{\mathrm{cm}}^{-1}$ (698 K, 24 h; Index 15) and 7.23(±0.17%) $\mathrm{mS}\;{\mathrm{cm}}^{-1}$ (723 K, 24 h; Index 30), indicating the discovery of a robust and reproducible synthesis regime.

The BO campaign was terminated after 32 experiments once a stable high‐performance region was identified, with multiple nearby conditions yielding reproducibly high ionic conductivities. At this stage, the expected improvement diminished significantly, indicating convergence of the optimization. Further iterations were therefore not pursued, also considering the experimental cost associated with each synthesis.

**FIGURE 1:**
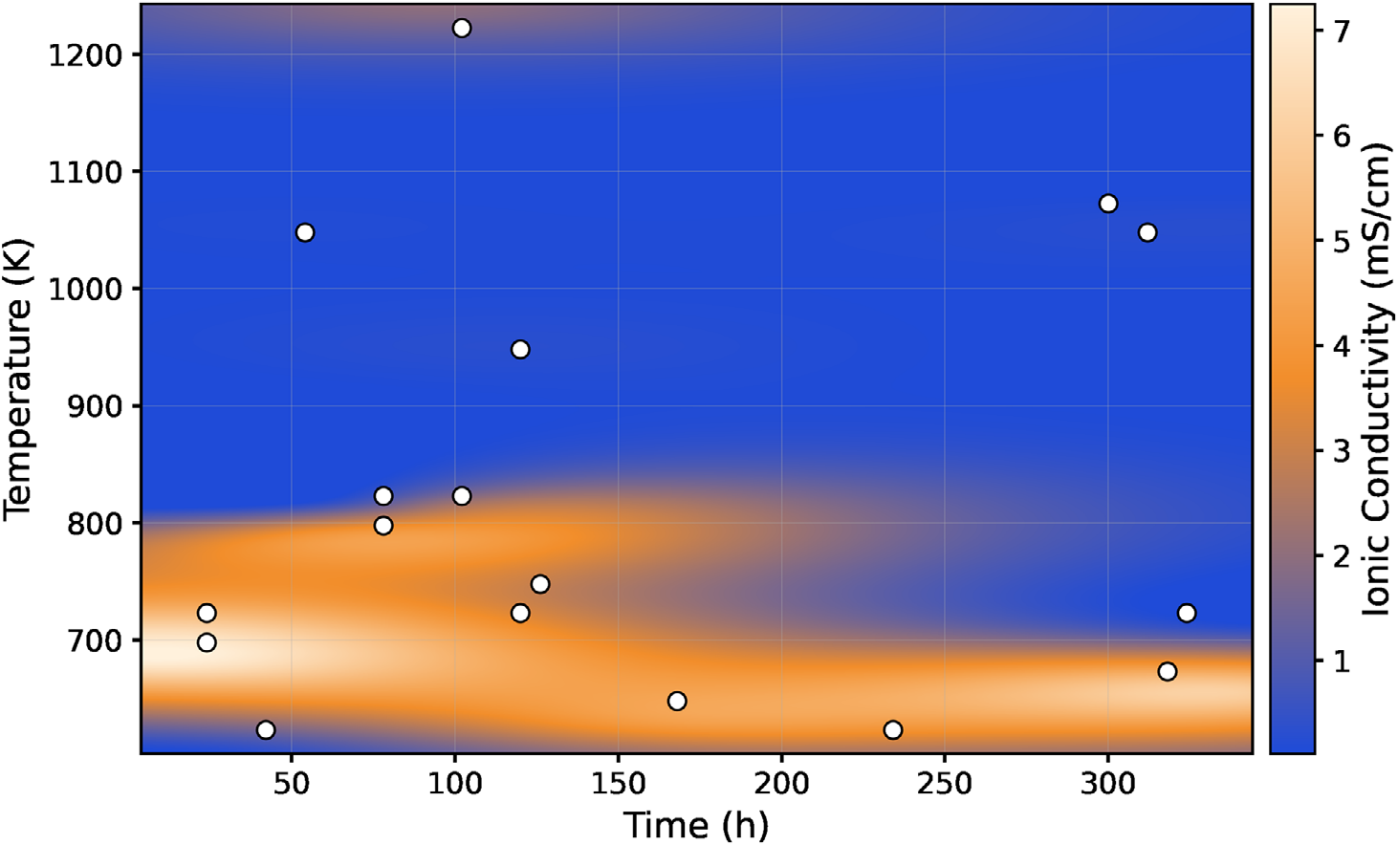
A map of the predicted ionic conductivity as a function of synthesis temperature and time, generated by the final GP surrogate model after optimization. This visualization depicts the model's prediction after the Bayesian Optimization process identified the synthesis conditions that yield the maximum ionic conductivity. The white circles indicate the specific temperature and time conditions of the experimentally synthesized samples used to train the model. The color scale corresponds to the predicted ionic conductivity, with warmer colors (orange) highlighting the region of optimal synthesis parameters identified by the model.

Figure 1 presents the model‐predicted landscape learned by the GP surrogate model after incorporating the experimental data that first reached ionic conductivity above 7 mS ${\mathrm{cm}}^{-1}$. The heatmap reveals a well‐defined region of fast ionic conductivity (yellow–red area) centered at temperatures from approximately 650 to 750 K and synthesis times of 24–48 h. In contrast, synthesis at temperatures above 850 K consistently yielded low conductivity, largely independent of annealing time. This demonstrates the BO framework's ability not only to efficiently identify optimal conditions but also to explore the parameter space, thereby avoiding unnecessary experiments in suboptimal regions. Given the strongly non‐monotonic dependence of ionic conductivity on synthesis temperature and time, identifying this narrow optimal window by exhaustive or random sampling would require substantially more experiments. BO of the ionic conductivity of ${\mathrm{Li}}_{7}{\mathrm{SiPS}}_{8}$ identified a synthesis protocol that employs a lower synthesis temperature (700 K) and a shorter dwell time (24 h), yielding samples with considerably higher conductivities. While BO itself does not impose a mechanistic model, the surrogate model captures correlations between synthesis parameters and performance. However, extracting physically meaningful causal relationships from this limited dataset remains challenging. Therefore, we formulated three hypotheses to explain the observed improvement. First, ${\mathrm{Li}}_{7}{\mathrm{SiPS}}_{8}$ exists in two polymorphs: the fast‐conducting *tetra*‐${\mathrm{Li}}_{7}{\mathrm{SiPS}}_{8}$ (2 mS ${\mathrm{cm}}^{-1}$) and a slower‐conducting *ortho*‐${\mathrm{Li}}_{7}{\mathrm{SiPS}}_{8}$ (0.1 mS ${\mathrm{cm}}^{-1}$). The orthorhombic modification is known to form at temperatures ≥800 K; thus, a synthesis temperature of 700 K should preferentially stabilize the tetragonal polymorph [14]. Second, all ${\mathrm{Li}}_{7}{\mathrm{SiPS}}_{8}$ powders contain varying amounts of amorphous side phases, which can impede ${\mathrm{Li}}^{+}$ transport. Third, the optimized protocol may alter the (micro‐)structure, producing larger grains and/or reducing grain‐boundary resistance, slightly influencing the crystal structure. All of these effects are known to enhance ionic conductivity [37, 38]. To test these hypotheses, we performed quantitative PXRD on all samples to determine their phase fractions. Six representative specimens were then selected for a comprehensive structural investigation using synchrotron PDF analysis, 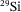
‐ and 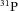 solid‐state NMR, 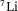
‐PFG‐NMR, SEM and EDX, and Raman spectroscopy. This multi‐modal approach was chosen in order to correlate the observed conductivity enhancements with specific changes in phase composition, local atomic ordering, and micro‐structural features, the results of which are discussed in the following.

### 2.2 Origins of the Increased Ionic Conductivity of ${\mathrm{Li}}_{7}{\mathrm{SiPS}}_{8}$

**FIGURE 2:**
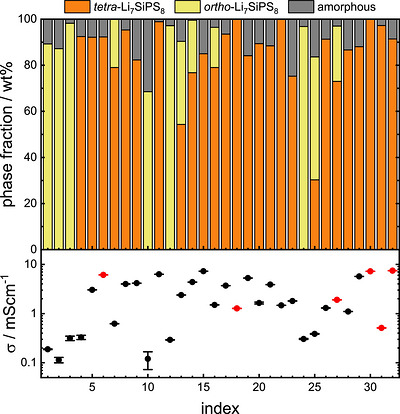
Phase fractions determined by laboratory PXRD as well as the corresponding ionic conductivities of all samples studied in this project. The tetragonal and orthorhombic ${\mathrm{Li}}_{7}{\mathrm{SiPS}}_{8}$ phase fractions are depicted in orange and yellow respectively, while the amorphous phase fraction is shown in gray. The ionic conductivity values of the studied subset are depicted in red.

The phase compositions and respective ionic conductivities of all samples are summarized in Figure 2. The phase compositions were determined by quantitative laboratory PXRD measurements using an internal Si standard, a method, whose applicability to the ${\mathrm{Li}}_{7}{\mathrm{SiPS}}_{8}$ system has already been reported [14, 35, 39]. The corresponding Rietveld fits and crystallographic information can be found in the Supporting Information (cf. Figures S1–S11 and Tables S2–S12). The ionic conductivities of all samples were determined by EIS, with corresponding data being shown in the Supporting Information as well (cf. Figures S12–S18). The samples are ordered by an arbitrary index used during the optimization. The exact synthesis parameters are tabulated in Table S1.

The synthesized samples each consist of 2–3 different phases, and the best‐performing samples exhibit ionic conductivities above 7 mS ${\mathrm{cm}}^{-1}$. This is a significant increase in comparison with the values initially reported in literature (2 mS ${\mathrm{cm}}^{-1}$) [14, 35]. It is clearly visible that samples containing large amounts of *ortho*‐${\mathrm{Li}}_{7}{\mathrm{SiPS}}_{8}$ and/or amorphous phase exhibit poor ionic conductivities. In general, the presence of the *ortho*‐${\mathrm{Li}}_{7}{\mathrm{SiPS}}_{8}$ phase seems to lower the ionic conductivity. Samples consisting of only the *tetra*‐${\mathrm{Li}}_{7}{\mathrm{SiPS}}_{8}$ phase and the amorphous phase seem to perform mostly, but not necessarily, better. There are some samples that show very comparable phase compositions (e.g., indices 4, 5, and 6), but vastly different ionic conductivities (0.33(±3.3%)–6.1(±1.1%) $\mathrm{mS}\;{\mathrm{cm}}^{-1}$). The ionic conductivity appears to depend only partly on the exact phase composition.

For further, more detailed analysis, a representative subset of 6 fast‐ and slow‐conducting samples was chosen. Samples with the indices 6, 30, and 32 are fast ion‐conducting ($\sigma_{ion}\approx$ 7 mS ${\mathrm{cm}}^{-1}$), while indices 18, 27, and 31 represent poor ion conduction ($\sigma_{ion}\approx$ $1\;\text{mS}\;{\text{cm}}^{-1}$).

**FIGURE 3:**
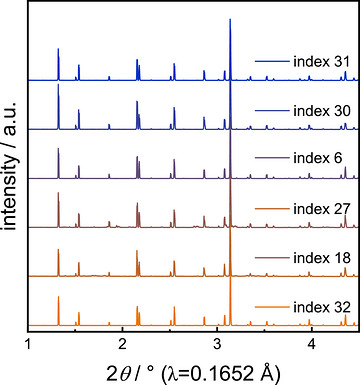
Synchrotron PXRD patterns of the studied subset in the 2θ range of 1
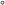
–4.5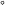. The full diffractograms are available in Figure S27.

**FIGURE 4:**
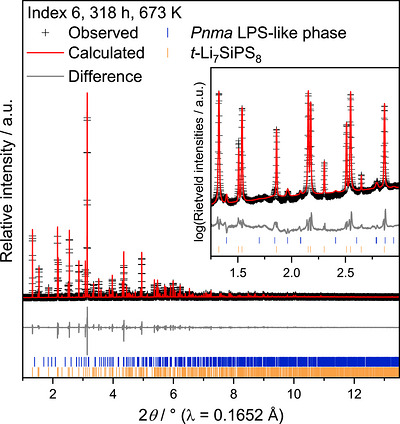
Rietveld refinement of the synchrotron XRPD data of the sample with the index 6. The graph shows the whole diffractogram with a linear *y*‐axis. The inset shows the logarithmic intensities plotted between 1.25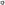 and 3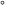 2θ to make the reflections of a strongly disordered Pnma ${\mathrm{Li}}_{3}{\mathrm{PS}}_{4}$ (LPS)‐like phase with highly anisotropic peak broadening visible.

The synchrotron PXRD patterns on the studied sample subset are shown in Figure 3. Using both synchrotron and laboratory PXRD data yields identical phase compositions (cf. Figure S28 and Tables S16–S17). All samples show reflections assigned to *tetra*‐${\mathrm{Li}}_{7}{\mathrm{SiPS}}_{8}$, while sample index 27, in addition, contains small amounts of *ortho*‐${\mathrm{Li}}_{7}{\mathrm{SiPS}}_{8}$. However, if the *y*‐axis is plotted logarithmically instead of linearly, previously not visible reflections appear (cf. Figure 4). These new reflections exhibit a pronounced anisotropic peak broadening, making them practically invisible while plotting the diffractograms with a linear *y*‐axis. Rietveld refinements reveal that they belong to a heavily distorted, orthorhombic ${\mathrm{Li}}_{3}{\mathrm{PS}}_{4}$‐like phase (around 1–3 wt%). This is visible in all samples except index 27, which contains *ortho*‐${\mathrm{Li}}_{7}{\mathrm{SiPS}}_{8}$ as the reflections overlap (cf. Figures S28). However, since both slow‐ and fast‐conducting samples contain this distorted ${\mathrm{Li}}_{3}{\mathrm{PS}}_{4}$‐like phase, no direct correlation with ionic conductivity and phase composition can be drawn. To obtain more information on the short‐range order in the sample, which might influence ionic conductivity, we performed total‐scattering PDF analysis. The PDFs are shown in Figures S29–S34. PDF analysis of the samples revealed no significant differences. A detailed discussion of the fitting procedure is provided in the Supporting Information.

**FIGURE 5:**
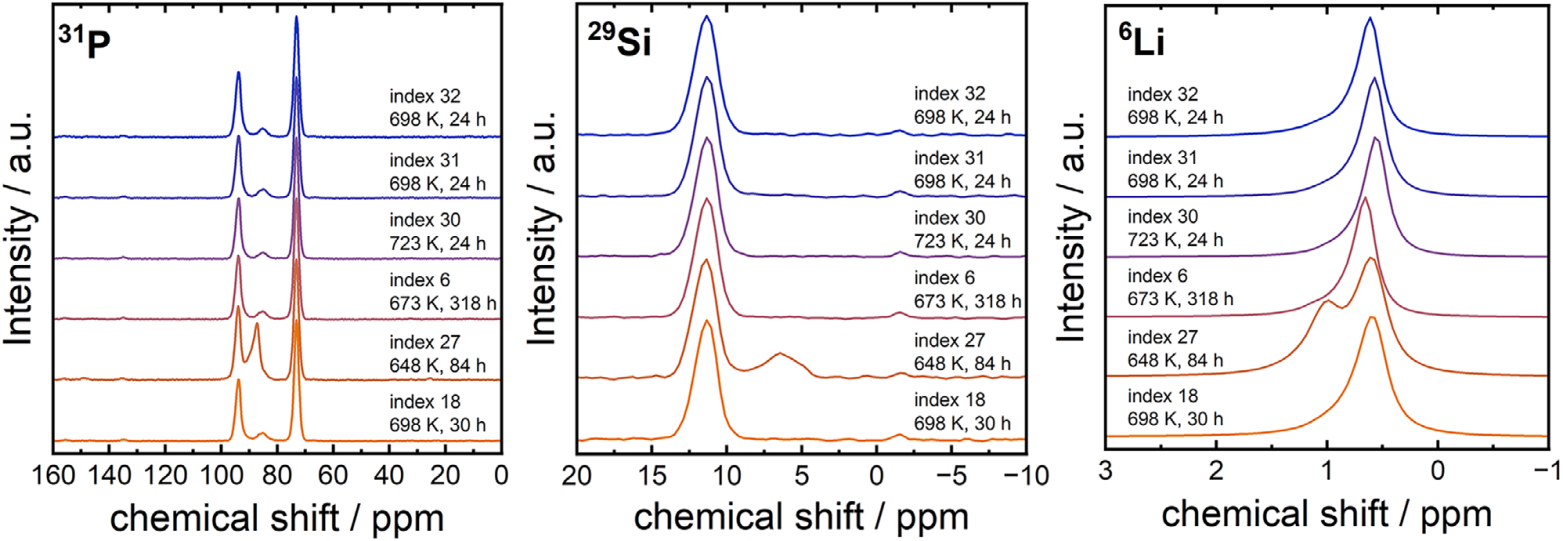
^31^P, ^29^Si, and ^6^Li solid‐state MAS‐NMR spectra of the chosen sample subset. The rotors were spun at 10 kHz.

The ^31^P, ^29^Si, and ^6^Li solid‐state MAS‐NMR spectra of the sample subset are shown in Figure 5. All spectra show expected signals corresponding to the determined phase composition of each sample. With the exception of one sample, the ^31^P and ^29^Si NMR spectra of all samples are nearly identical. The same signals as reported by Harm et al. and Calaminus et al. for tetragonal ${\mathrm{Li}}_{7}{\mathrm{SiPS}}_{8}$ were found [14, 35]. In the ^31^P NMR, three signals at 94, 85, and 73 ppm are present, falling well within the chemical shift range expected for ${\mathrm{PS}}_{4}$ tetrahedra [40, 41]. Hence, the three signals were attributed to the phosphorous at the 4d Wyckoff position, the amorphous phase, and the 2b Wyckoff position, respectively. The ^31^P spectrum of sample index 27 exhibits a signal at 87.5 ppm, which stems from the *ortho*‐${\mathrm{Li}}_{7}{\mathrm{SiPS}}_{8}$ phase that can also be seen in the PXRD measurements (cf. Figure S9) [14]. Table S14 shows the amorphous phase fraction calculated from the integrated ^31^P NMR signals. In contrast to the amorphous phase fractions from the Rietveld refinements, the values are much more similar to each other. The mean value (4.2 wt%) is slightly higher than that from Rietveld refinements (3.4 wt%), and the standard deviation is much lower (0.52 vs. 4.1 wt%). The internal standard method is less accurate than NMR for estimating the amorphous phase content, since several factors beyond the amorphous phase content contribute to the background. However, using NMR to quantify the amorphous phase fraction requires an idea of its composition to convert the atom% obtained from integrating NMR signals into a weight fraction.

All ^29^Si NMR spectra show a strong signal at 11 ppm that is associated with the ${\mathrm{SiS}}_{4}$ tetrahedra found in the *tetra*‐${\mathrm{Li}}_{7}{\mathrm{SiPS}}_{8}$ structure and a weak signal at −1.5 ppm that is attributed to the amorphous phase [14, 19, 42]. The ^29^Si spectrum of sample index 27 shows another signal at around 6.5 ppm that, again, is caused by the *ortho*‐${\mathrm{Li}}_{7}{\mathrm{SiPS}}_{8}$ side phase [14]. Some variation can also be seen in the ^6^Li NMR (cf. Figure 5). The ^6^Li spectra show a slight difference in the signal positions (between 0.57 and 0.67 ppm) as well as signal width. This variation in chemical shift might hint at small variations in the crystal structure and lithium environments across samples. However, no correlation between the chemical shifts and ionic conductivities of each sample is visible. Some samples exhibit a small shoulder around 1 ppm. The shoulder is most pronounced in the sample indexed 27 with the *ortho*‐${\mathrm{Li}}_{7}{\mathrm{SiPS}}_{8}$ side phase. While the main signal is due to the tetragonal ${\mathrm{Li}}_{7}{\mathrm{SiPS}}_{8}$ phase, the shoulder is assigned to the *ortho*‐${\mathrm{Li}}_{7}{\mathrm{SiPS}}_{8}$ or a chemically very similar amorphous side phase. ^7^Li NMR spectra of the same samples are shown in Figure S26. To exclude sample decomposition due to moisture, ^1^H NMR spectra were recorded as well (Figure S26). Again, the ^1^H spectra look very similar and contain roughly the same amount of protons (cf. Table S13), hence no significant influence on ionic conductivity by sample decomposition is expected.

**FIGURE 6:**
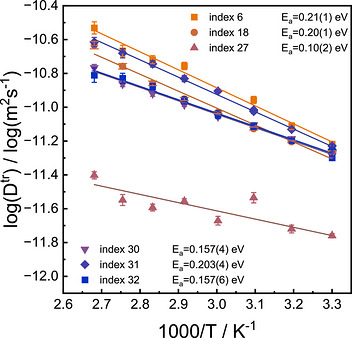
Arrhenius plots of the diffusion coefficients $D_{NMR}^{tr}$ obtained by ^7^Li PFG NMR of the studied samples and the obtained activation energies $E_{a}$ for diffusion.

To gain more detailed insights into the Li‐ion conduction mechanism, we used temperature‐dependent PFG NMR. Figure 6 visualizes the diffusion variables of the samples at different temperatures. The Arrhenius equation was used to calculate activation energies, which are shown in the graph legend. It can be seen that the samples with indices 27, 30, and 31 show significantly lower activation energies of 0.10(2), 0.157(6), and 0.157(4) eV, respectively, in comparison with the other samples, which have activation energies of around 0.2 eV. This is still slightly below the values given in the literature. However, due to different pulse spacings $\Delta_{NMR}$, a comparison is difficult [14]. Despite the presence of the *ortho*‐${\mathrm{Li}}_{7}{\mathrm{SiPS}}_{8}$ phase and slow ionic conductivity as measured by EIS, index 27 exhibits a surprisingly low (PFG NMR) activation energy and, at the same time, slower ion diffusion. However, its data points also have larger errors than the other measurements, probably due to multiple phases or a lower overall diffusion in sample 27. The activation energies from PFG NMR are comparable to those obtained by temperature‐dependent EIS (cf. Figure S19) of the respective samples. In the EIS measurements, index 27 has a higher activation energy (0.166(3) eV), while index 31 has a lower‐than‐expected activation energy (0.089(5) eV). The obtained tracer diffusion coefficient and the gradient pulse spacing $\Delta_{NMR}$ can be used to estimate the three‐dimensional isotropic diffusion radius r or the probed diffusion length, respectively, using Equation (1) [43, 44]

(1) $$r_{\mathrm{rms}}=\sqrt{6D_{\mathrm{NMR}}^{\mathrm{tr}}\cdot \Delta_{\mathrm{NMR}}}$$

In this equation, $D_{NMR}^{tr}$ is the tracer diffusion coefficient determined by NMR and $\Delta_{NMR}$ is the time between field gradient pulses. The calculated values can be seen in Table S15. The obtained r values of the samples at RT vary between 1250 and 1348 nm, with the exception of index 27, which has a significantly smaller r value of 722 nm. All obtained diffusion radii are substantially larger than the ≈ 200 nm reported by Harm et al. [14] The larger isotropic diffusion radii hint at a fast diffusion process that is no longer confined to small domains.

From the diffusion coefficients, it is also possible to calculate a bulk ionic conductivity $\sigma_{NMR}$ using the Nernst–Einstein equation (cf. Equation 2), where $D_{NMR}^{tr}$ is the tracer diffusion coefficient determined by NMR, n is the charge carrier concentration, z is the charge of the charge carrier, e is the elementary charge, $k_{B}$ is the Boltzmann constant, T is the temperature, and $H_{R}$ is the Haven ratio [45, 46, 47, 48]. The calculated conductivities for a Haven ratio of $H_{R}=1$ (meaning uncorrelated ion motion) can be found in Table S15. The values can be considered a lower limit limit for the conductivity, since other LGPS‐type electrolytes exhibit a correlated jump process ($H_{R}<1$) [45].

(2) $$\sigma_{\mathrm{NMR}}=\frac{D_{\mathrm{NMR}}^{\mathrm{tr}}nz^{2}e^{2}}{k_{b}TH_{R}}$$

**FIGURE 7:**
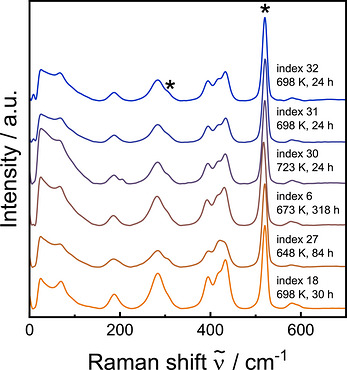
Raman spectra of the subset of investigated samples. The samples exhibit the typical vibrations expected for ${\mathrm{PS}}_{4}$ and ${\mathrm{SiS}}_{4}$ tetrahedra between 393 and 433 ${\mathrm{cm}}^{-1}$. The bands marked with an asterisk at 520 and 305 ${\mathrm{cm}}^{-1}$ stem from the silicon standard.

The Raman spectra of the representative dataset are shown in Figure 7. The Raman data were acquired using Silicon as an internal reference. Therefore, the main stretching vibration for the Si standard is visible in all spectra at 520 ${\mathrm{cm}}^{-1}$, which was used to reference all spectra [49, 50, 51]. The silicon causes a second, much weaker signal at ≈ 305 ${\mathrm{cm}}^{-1}$, which is visible as a small shoulder in all samples [52, 53]. The observed spectra exhibit a high degree of similarity and align with the expected values from literature. In the high‐frequency region above 150 ${\mathrm{cm}}^{-1}$, the most prominent bands at 393, 415, and 433 ${\mathrm{cm}}^{-1}$ can be attributed to Si−S and two distinct P−S vibrations in the *tetra*‐${\mathrm{Li}}_{7}{\mathrm{SiPS}}_{8}$ phase, respectively [14, 19, 35, 42, 54, 55, 56]. Notably, sample 27 displays a band broadening at around 420 ${\mathrm{cm}}^{-1}$, caused by an additional band due to the P−S vibration of the orthorhombic ${\mathrm{Li}}_{7}{\mathrm{SiPS}}_{8}$ phase. Furthermore, the broad signals at 280 and 580 ${\mathrm{cm}}^{-1}$ are also associated with P−S vibrations, whereas the peak at 185 ${\mathrm{cm}}^{-1}$ is due to Li−S vibrations [14, 35, 55, 56]. The spectrum of index 30 shows an additional band at ≈215 ${\mathrm{cm}}^{-1}$, which is not present in the other spectra. The additional band was assigned to residual sulfur, which might not have fully reacted during synthesis [57, 58]. Lastly, the peak at 70 ${\mathrm{cm}}^{-1}$ in the low frequency range might be another vibrational or rotational mode, or the so‐called boson peak, which is typical for amorphous or highly disordered materials [59, 60]. In summary, all Raman spectra show the expected vibrations concurrent with ${\mathrm{Li}}_{7}{\mathrm{SiPS}}_{8}$. While there are some differences between the spectra, these are very minor, thus not allowing any correlation with ionic conductivity.

Scanning electron microscopy (SEM) images and energy dispersive x‐ray spectroscopy (EDX) maps are shown in Figures S21–S25. SEM and EDX were performed to investigate the microstructure and compositions of the samples, since these factors are also known to influence the ionic conductivity [61, 62]. The morphology and secondary particle size distribution of all samples are very similar. As described in the literature, the samples consist of crystalline particles with average grain sizes of 5–30 μm that are embedded in an amorphous, glassy matrix [14, 35]. However, the method does not allow any conclusions to be drawn about the primary particle size. Similarly, EDX confirmed the stoichiometry, while the EDX maps show an even distribution of Si, P, and S throughout all samples. A more detailed discussion is available in the Supporting Information.

The general understanding is that ${\mathrm{Li}}_{7}{\mathrm{SiPS}}_{8}$ is a glassy ceramic consisting of a crystalline tetragonal phase and an amorphous phase [14, 35]. This matches with the Raman and NMR data. However, high‐resolution synchrotron XRPD data reveal the presence of a heavily distorted orthorhombic ${\mathrm{Li}}_{3}{\mathrm{PS}}_{4}$ phase instead of an amorphous phase. This explanation is also consistent with the Raman and ^31^P NMR. Notably, the signal associated with the amorphous phase appears at the same chemical shift as the orthorhombic ${\mathrm{Li}}_{3}{\mathrm{PS}}_{4}$ phase. It is, however, much broader, pointing to lower crystallinity as would be expected for a heavily disordered phase.

After our detailed analysis of the collected data, we revisit our three working hypotheses to explain why ${\mathrm{Li}}_{7}{\mathrm{SiPS}}_{8}$ shows increased ionic conductivity after applying the improved synthesis procedure obtained using BO. Our quantitative PXRD refinements show that samples containing a significant amount of *ortho*‐${\mathrm{Li}}_{7}{\mathrm{SiPS}}_{8}$ exhibited slower ion conduction. However, a high *tetra*‐${\mathrm{Li}}_{7}{\mathrm{SiPS}}_{8}$ content alone does not guarantee fast ion conduction. For instance, sample 18 is 100 wt% crystalline *tetra*‐${\mathrm{Li}}_{7}{\mathrm{SiPS}}_{8}$ yet displays only 1.28(±0.62%) $\mathrm{mS}\;{\mathrm{cm}}^{-1}$ at RT, far below the best values obtained in this study. This disproves the first hypothesis that the ionic conductivity is solely caused by an increase of the relative fraction of the fast‐conducting *tetra*‐${\mathrm{Li}}_{7}{\mathrm{SiPS}}_{8}$ versus the orthorhombic polymorph. The hypothesis that a reduction in amorphous phase fraction could be responsible also seems to be invalid as the amorphous component, quantified by Rietveld analysis, does not correlate with conductivity. Samples 6 (7.7 wt% amorphous), 30 (0 wt%), and 32 (8.6 wt%) all achieve essentially the same conductivity (≈ 7 mS ${\mathrm{cm}}^{-1}$). Thus, while amorphous phases are known to impede ${\mathrm{Li}}^{+}$ transport in some SEs [14, 63, 64], their presence does not appear to be the dominant factor influencing the ionic conductivity of ${\mathrm{Li}}_{7}{\mathrm{SiPS}}_{8}$ under the examined conditions.

SEM images reveal similar grain (secondary particle) sizes across the entire subset, and 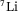 PFG NMR data show similar isotropic diffusion radii for all studied samples, meaning that subtle differences in the microstructure, which can affect ${\mathrm{Li}}^{+}$ diffusion, are unlikely to be responsible for the conductivity increase. Likewise, the ^31^P and ^29^Si MAS NMR spectra as well as Raman data show only minor differences with all expected NMR signals and vibrations of the ${\mathrm{SiS}}_{4}$ and ${\mathrm{PS}}_{4}$ tetrahedra present. Only the 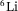 and 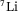 NMR spectra show slight differences in the chemical shifts, which might hint at a slightly different Li substructure across the samples, but they do not follow an obvious trend. Further analysis is required to confirm this hypothesis.

Summarizing, none of the probed individual parameters (phase composition, amorphous content, grain size, local atomic ordering, or vibrational signatures) exhibit a systematic relationship with the >350% increase in the ${\mathrm{Li}}_{7}{\mathrm{SiPS}}_{8}$ RT ionic conductivity. This result, however, is not a methodological shortcoming but rather reflects the complexity of the ${\mathrm{Li}}_{7}{\mathrm{SiPS}}_{8}$ system. We suggest that the absence of a conclusive correlation between the ionic conductivity and our measured data is either caused by an as‐yet unidentified factor governing the ${\mathrm{Li}}^{+}$ diffusion process or due to a complex interplay of the studied factors at work. Further work using, among others, neutron‐based techniques or/and first‐principle methods is required in order to elucidate the exact ${\mathrm{Li}}^{+}$ diffusion mechanism present in the ${\mathrm{Li}}_{7}{\mathrm{SiPS}}_{8}$ system. However, it is very difficult to determine exactly how the various factors influence ionic conductivity and how they correlate with one another. This is probably only possible with machine learning algorithms that require large data sets of several hundred samples.

## 3 Conclusion

In this study, we demonstrated that ML‐driven optimization, specifically BO, can be harnessed to significantly improve the ionic conductivity of sulfidic SEs. By systematically optimizing the synthesis procedure, we successfully and reproducibly increased the ionic conductivity of $Li_{7}{\mathrm{SiPS}}_{8}$ by ≈ 350% (from 2 to over 7 mS ${\mathrm{cm}}^{-1}$ at RT). Concurrently, we achieved a 20% lower synthesis temperature (from 798 to 698 K) and shortened the reaction duration by 76% (from 100 to 24 h), thereby delivering a more energy‐efficient synthesis. Because BO does not require an explicit mechanistic model of the relationship between synthesis variables and performance, we complemented the optimization with a thorough analysis of six representative samples using synchrotron PXRD and PDF analysis, MAS and PFG solid‐state NMR, and Raman spectroscopy. Despite our exhaustive efforts, no single dominant descriptor governing the conductivity enhancement could be identified within the probed parameter space, with all samples showing only minor differences, following no clear trend. Nevertheless, by using BO, we succeeded in optimizing the complex ${\mathrm{Li}}_{7}{\mathrm{SiPS}}_{8}$ system without explicit mechanistic insight. Since BO models the target property probabilistically as a function of the input parameters, the same strategy can be readily transferred to other optimization problems in SSB research. Beyond our two‐dimensional parameter space (temperature and time) BO processes might include higher‐dimensional feature spaces with parameters such as precursor history (e.g., milling conditions, aging) [65], microstructural descriptors (e.g., particle size distributions), kinetic parameters (e.g., heating‐ and cooling rates), or even in situ/operando features derived from characterization techniques. By including these parameters other important SE properties (besides ionic conductivities) like microstructure, activation energies, or interphases may be optimized. Our findings highlight the power of data‐driven methods for developing next‐generation high‐performance SEs.

## Conflicts of Interest

Dr. Yinghan Zhao declares that this work was done prior to him joining Amazon Development Center Germany GmbH. The other authors declare no conflicts of interest.

## Supporting information

The Supporting Information is available free of charge at [LINK]. The Supporting Information contains: sample overview, experimental section, details of the BO, quantitative phase analysis, EIS, SEM/EDX, ^7^Li and ^1^H MAS NMR, phase fractions determined by NMR, ionic conductivities and isotropic ion diffusion radii calculated from PFG NMR detailed synchrotron PXRD and PDF data.
The authors have cited additional references within the Supporting Information [67, 68, 69, 70, 71, 72, 73, 74, 75, 76, 77, 78, 79, 80, 81, 82, 83, 84, 85, 86, 87, 88, 89, 90].
**Supporting File 1**: anie72629‐sup‐0001‐SuppMat.pdf.

## Data Availability

The data that support the findings of this study, including the code employed for BO, are openly available in the Edmond repository at https://doi.org/10.17617/3.PA07LU.
