# Supplementary material for: Optimize Before You Synthesize—Enhancing the Ionic Conductivity of Li7SiPS8 Using Bayesian Optimization
Source: Angew Chem Int Ed Engl. 2026 May 23;65(30):e5778118. doi: 10.1002/anie.5778118 (PMC13383069; doi:10.1002/anie.5778118)
Supplement: Supplementary file 1 — The Supporting Information is available free of charge at [LINK]. The Supporting Information contains: sample overview, experimental section, details of the BO, quantitative phase analysis, EIS, SEM/EDX, 7Li and 1H MAS NMR, phase fractions determined by NMR, ionic conductivities and isotropic ion diffusion radii calculated from PFG NMR detailed synchrotron PXRD and PDF data. The authors have cited additional references within the Supporting Information [67, 68, 69, 70, 71, 72, 73, 74, 75, 76, 77, 78, 79, 80, 81, 82, 83, 84, 85, 86, 87, 88, 89, 90]. Supporting File 1: anie72629‐sup‐0001‐SuppMat.pdf. [file ANIE-65-e5778118-s001.pdf]

Supporting Information: Optimize before you Synthesize — Enhancing the Ionic Conductivity of Li_7SiPS_8 using Bayesian Optimization

Lucas G. Balzat,[†,‡,¶] Robert Calaminus,[†,‡,¶] Yinghan Zhao,[§,‖] Kristina Gjorgjevikj,[†,⊥] Igor Moudrakovski,[†] Simon Krause,[⊥,†] Arnd Koeppe,[§] Britta Nestler,[§] and Bettina V. Lotsch[*,†,‡]

†*Department of Nanochemistry, Max Planck Institute for Solid State Research, Heisenbergstraße 1, 70569 Stuttgart, Germany*

‡*Department of Chemistry, Ludwig-Maximilian-University Munich, Butenandtstraße 5-13, 81377 Munich, Germany*

¶*These authors contributed equally to this work*

§*Institute for Applied Materials - Microstructure Modelling and Simulation, Karlsruhe Institute of Technology, Straße am Forum 7, 76313 Karlsruhe, Germany*

‖*Current Address: Amazon Development Center Germany GmbH, Krausenstraße 38, 10117 Berlin, Germany*

⊥*Institute for Inorganic Chemistry II, Ulm University, Albert-Einstein-Allee 11, 89081 Ulm, Germany*

E-mail: b.lotsch@fkf.mpg.de

Sample overview

Table S1: Overview of the synthesis parameters, resulting ionic conductivities and the relative pellet densities of all prepared samples. For the calculated pellet densities, the sample densities from XRPD were used.

index	T_{syn} [K]	t_{syn} [h]	σ_{ion} [mScm^{-1}]	pellet density [%]
1	1048	54	0.188	95.44
2	1073	300	0.114	89.74
3	948	120	0.315	84.50
4	723	324	0.329	77.79
5	748	126	3.030	81.72
6	673	318	6.100	90.66
7	821	78	0.622	98.31
8	723	120	3.990	85.81
9	798	78	4.140	86.36
10	896	42	0.120	94.84
11	723	24	6.290	93.03
12	1048	312	0.292	84.78
13	623	234	2.380	89.82
14	648	168	4.360	91.85
15	698	24	7.250	92.33
16	648	318	1.668	93.74
17	673	24	3.673	90.63
18	698	30	1.275	92.31
19	798	24	5.270	81.65
20	748	24	1.640	86.15
21	698	318	3.880	79.64
22	698	30	1.577	92.31
23	698	18	1.828	91.08
24	998	24	0.305	77.74
25	698	168	0.386	93.39
26	773	24	1.209	87.79
27	648	84	1.909	89.56
28	698	24	1.207	79.96
29	723	24	5.690	86.95
30	723	24	7.230	91.28
31	698	24	0.510	84.82
32	698	24	7.430	83.81

Experimental

Synthesis

In order to achieve a consistent synthesis, 10 g of a precursor mixture were prepared by ball-milling stoichiometric amounts of Li_2S (99.98%, Aldrich), Si (99.999%, Alfa Aesar), red P (≥97%, Merck), and S (sublimated in vacuo, Grüssing). In order to counter sulfur loss due to sublimation during synthesis, a 5 wt% excess of S was added. A Retsch PM200 planetary ball mill with a 50 mL ZrO_2 jar and 20 zirconia balls (10 mm diameter) was used to blend the starting materials. The milling program consisted of 3 steps. In the first step, the mixture was milled for 1 h (5 min milling, 1 min pause) at 350 rpm. Afterwards the mixture was hand ground in an agate mortar for 5 minutes, before it was milled again for 1 h (3 min milling, 3 min pause) at 350 rpm. Throughout the project, the same precursor mixture was used for all synthesized Li_7SiPS_8 samples.

For synthesis, approximately 250-500 mg of the precursor mix was transferred into glassy carbon crucibles, which were subsequently sealed in a quartz glass ampule under vacuum using an oxyhydrogen torch. The ampules were then put into a tubular furnace. For consistency in synthesis, the same two furnaces were used for all syntheses throughout the project. The ampules were then heated at a rate of 50 K h^{-1} to a target temperature and held there for a fixed time. The target temperatures and dwell times were obtained from Bayesian optimization and are listed in Table S1. Afterwards, the furnace was switched off, and the samples were left to cool down naturally. All products were obtained as yellow to pale orange powders. Due to the moisture sensitivity of Li_2S and the products, all sample handling was performed under inert conditions inside an argon-filled glove box (MBraun, $O_2 < 0.1$ ppm, $H_2O < 1$ ppm) or in argon-filled containers at all times. In the following, the samples are only referred to by their index (cf. Table S1).

Electrochemical Impedance Spectroscopy (EIS)

For EIS, approximately 30 mg of sample material were ground in an agate mortar and then compacted into 5 mm diameter pellets using uniaxial cold-pressing ($p \approx 1$ GPa). The pellets were then sputtered with platinum using a Quorum Q150 GB sputter coater to improve contact between the pellets and the electrodes. The coated pellets were then put into RHD instruments TSC SW closed measurement cells. The pressure in the measurement cells was set to $\approx$ 650 kPa. The cells were then connected to an RHD Instruments Microcell HC cell stand, which allowed controlling the measurement temperature. EIS was measured in an ion-blocking, two-electrode setup using an Ivium Compactstat.h potentiostat between 1 MHz-1 Hz using an excitation voltage of 10 mV.

The ionic conductivity σ was extracted by means of equivalent circuit fitting using resistors, capacitors, and constant phase elements (CPEs). In some cases, inductors were used to compensate for cable induction. The capacities of the CPEs were calculated using the Brug formula and used to assign processes to the semicircles.[S1] Bulk and grain boundary contributions ($C_{Brug} \leq 10^{-10}$ F) were not resolvable in all cases, hence only total conductivities are reported here. Processes with larger capacities were also fitted, but not used in the calculation of ionic conductivities.[S2]

Temperature-dependent measurements were carried out between 25 °C and -20 °C or -15 °C (it was not always possible to cool down completely) in 5° C steps. The pellets were held at the different temperatures for 1 h to allow the system to equilibrate to the temperature. The activation energy E_a was obtained by fitting the temperature-dependent conductivities to the modified Arrhenius equation $\sigma T = \sigma_0 e^{\frac{-E_a}{k_b T}}$ using the temperature T, the pre-exponential factor σ_0, and Boltzmann´s constant k_b.[S3]

Scanning electron microscopy & energy-dispersive x-ray spectroscopy

Scanning electron microscopy (SEM) images and energy-dispersive X-ray (EDX) spectra were recorded on a Zeiss Merlin with an Ultim Extreme EDX detector. Acceleration voltages of

1.5 kV and 5 kV for imaging and for EDX measurements, respectively, were used. The samples were not sputtered. The samples had brief contact with air during sample transfer into the electron microscope.

Laboratory Powder X-ray Diffraction (PXRD)

For PXRD, all samples were ground in an agate mortar, loaded into glass capillaries (∅ = 0.5 mm, Hilgenberg), and subsequently sealed under argon. The PXRD patterns were obtained using a Stoe STADI P diffractometer (Ag $K_{\alpha 1}$ radiation, Ge-(111) monochromator, Mythen 1K Detector) in Debye–Scherrer geometry. For quantitative PXRD, a defined amount of ball-milled silicon single crystals (Alfa Aesar, 99.999%) was added as an internal standard to determine the phase fractions and the amount of amorphous side phase (sample:Si ratio = 2:1). (Quantitative) Rietveld refinements were carried out with the software Topas v. 6.[S4]

Synchrotron PXRD & PDF

Synchrotron PXRD and PDF data were obtained through the services of the company Momentum Transfer GmbH.[S5] High-resolution synchrotron X-ray diffraction and total scattering experiments[S6,S7] were performed at the ID31 beamline at the European Synchrotron Radiation Facility (ESRF). The measurements were performed using a high-throughput sample holder with Kapton windows and approx. 1 mm-thick cylindrical slots that were filled with the respective sample powder. Samples were measured in transmission geometry with an incident X-ray energy of 75.051 keV ($\lambda = 0.16520$ Å), and intensities were recorded using a Pilatus CdTe 2M detector (1679×1475 pixels, 172×172 μ^2 each) positioned with the incident beam in the corner of the detector. The distance between the sample and the detector was approximately. 1.5 m for the high-resolution and 0.3 m for the total scattering experiment. Multiple measurements of the empty well with polyimide windows were performed and summed to improve statistics for background subtraction.

Geometry calibration for the measurements was done using a NIST SRM 660b (LaB_6) standard. Integration was performed using the software pyFAI, including flat-field, geometry, solid-angle, and polarization corrections. Invalid pixels were masked, and additional azimuthal outliers were automatically masked by the sigma-clipping integration algorithm. The summed background intensities were scaled respectively to each reference and sample measurement and subsequently subtracted. Topas v. 7[S4] was used to perform Rietveld refinements to determine the instrument profile contribution and correct for offset errors including parallax.[S8] This was done by fitting the structure with the reference lattice parameters fixed to the measurement.

Preliminary PDF data were automatically processed using the program PDFgetX3[S9–S11] fo range of Q_{max} values. The data were subsequently reprocessed using a Lorch modification function[S12] to suppress termination effects and contributions from high-frequency noise. Small-angle scattering intensities were extrapolated from $Q_{min} \sim 0.3$ Å^{-1} to 0. Compositional details were input into PDFgetX3 for data processing.

Sample handling

The sample containers were opened in an argon-filled glove box (O_2 and H_2O levels below 0.1 ppm). In the glove box, the samples were loaded into the sample holder. The sample holder was transferred to the beamline ID31 in a closed container under argon. The container was opened 10 min before the measurements, and the sample holder was kept under argon (2 hours) between the TS-PDF and the HT-XRPD measurements.

Solid-state NMR spectroscopy

Magic-angle spinning (MAS) experiments were performed in ZrO_2 spinners at a spinning speed of 10 kHz using a using a Bruker 4 mm triple-channel probe and a Bruker avance III spectrometer with a magnetic field of $B_0 = 9.4$ T. Tetramethyl silane ($Si(CH_3)_4$, $\delta_{iso} = 0.0$ ppm) was used to externally reference the ^{29}Si and ^{1}H NMR spectra. 85% phosphoric

acid (H_3PO_4, $\delta_{iso} = 0.0$ ppm) was used to reference the ^{31}P spectra. ^{6}Li and ^{7}Li spectra were referenced using an 1 M LiCl solution ($\delta_{iso} = 0.0$ ppm). For the quantification of the hydrogen content, a known amount of adamantane mixed with KBr was used as an external standard. The probe's background signal was measured and subtracted. The ^{7}Li pulsed field gradient (PFG) diffusion NMR experiments were carried out at 9.4 T using a diff60 single gradient diffusion probe that allows for pulsed field gradient g of up to 30 Tm^{-1} and temperatures between 303 and 373 K. All PFG experiments used a stimulated-echo pulse sequence.[S13] The diffusion coefficient D was obtained by fitting the experimental echo attenuation curves S(g, δ, Δ) with the Stejskal–Tanner equation[S14] (cf. eq. 1). The Stejskal–Tanner equation uses the ^{7}Li gyromagnetic ratio $\gamma = 1.398108$ HzT^{-1}, the duration of the pulsed field gradients δ, and the time interval between field gradient pulses Δ, which defines diffusion time. The measurements used a fixed δ value of 1 ms, a Δ value of 50 ms, while the gradient strengths were varied between 0.1 and 30 Tm^{-1}.

$$S(g, \delta, \Delta) = -\gamma^2 \delta^2 g^2 D(\Delta - \frac{\delta}{3}) \tag{1}$$

Raman spectroscopy

Raman spectroscopy was measured on a custom system comprising a 785 nm laser equipped with a Raman probe (Coherent TR-Probe 300 mW power at sample port, a steerable non-contact optic, and a flood light module as accessories) and connected to a spectrograph (Andor Kymera 328i, Andor iDUS 420 CCD detector). The samples were measured in the same capillaries as for PXRD analysis at 30 % laser power on the sample and at ambient temperature.

Additional information on Bayesian optimization

Bayesian optimization (BO) is a model-based, sequential optimization framework designed for the global optimization of expensive-to-evaluate black-box functions. It addresses the challenges posed by limited experimental budgets by constructing a probabilistic surrogate model of the objective function and using it to guide the selection of subsequent evaluation points in a data-efficient manner. In this study, BO was implemented using a Gaussian Process (GP) surrogate model with a Matérn 5/2 kernel, chosen for its flexibility in capturing moderately non-smooth response surfaces that are commonly encountered in experimental materials data.[S15] The acquisition function employed was Expected Improvement (EI), which balances exploration of uncertain regions of the parameter space with exploitation of synthesis conditions predicted to yield high performance.[S15] Model hyperparameters were optimized at each iteration by maximizing the marginal likelihood, and a Gaussian noise term was incorporated to account for experimental uncertainty in the conductivity measurements.

In our case, the uncertainties in ionic conductivity obtained from EIS fitting are consistently small ($\leq 1\%$) and were therefore not treated as explicit input to the BO model. Instead, a constant Gaussian noise term was included in the GP surrogate to account for experimental variability in a general manner. Given the much larger variation in conductivity across different synthesis conditions, explicit uncertainty propagation was not expected to affect the optimization outcome.

The BO procedure follows an iterative optimization loop. It begins with an initial set of observations $\{(x_i, y_i)\}$, where x_i denotes the input parameters and $y_i = f(x_i)$ represents the corresponding objective function values. The initial design may be informed by prior knowledge or expert intuition, or generated using space-filling strategies such as random sampling. Based on the accumulated data, the surrogate model is fitted and subsequently used to optimize the acquisition function, yielding the next candidate point x_{next}. This

candidate is then evaluated using the true experiment or simulation to obtain $y_{\text{next}} = f(x_{\text{next}})$. The new observation is added to the dataset, and the process is repeated until a predefined stopping criterion is met, such as convergence of the objective value, achievement of a target performance, or exhaustion of the experimental budget.

The iterative nature of BO can be viewed as a formalization of the decision-making process commonly employed by experienced researchers.[S15] In practice, scientific exploration often involves a balance between investigating uncertain or novel directions and refining conditions that have previously shown promise. BO provides a mathematically rigorous and data-driven framework for navigating this exploration–exploitation trade-off. By relying on statistical evidence from the surrogate model and the recommendations of the acquisition function, BO enables a more objective, potentially more globally effective search strategy, helping mitigate inherent human biases, such as overemphasizing conditions that have been successful in related but distinct systems.[S15]

At the core of Bayesian optimization lies the surrogate model, which approximates the relationship between input parameters and the target output while providing uncertainty estimates for its predictions. Among various probabilistic models, Gaussian Processes (GPs) are one of the most widely used and effective choices for surrogate modeling in BO.[S15] A GP defines a prior distribution over functions and assumes that the function values at any finite set of input points follow a joint Gaussian distribution. Formally, a function f is said to follow a Gaussian Process if, for any finite set of points $x_1, \ldots, x_N$, the vector $(f(x_1), \ldots, f(x_N))$ follows a multivariate Gaussian distribution.[S16] This is denoted as

$$f(x) \sim \mathcal{GP}(m(x), k(x, x')). \tag{2}$$

Here, $m(x)$ is the mean function, often assumed to be zero or a simple parametric form, representing the prior expectation of $f(x)$, while $k(x, x')$ is the covariance function (kernel), which encodes assumptions about the smoothness, characteristic length scales, and other

structural properties of the objective function by quantifying the correlation between function values at different input points.

Given a dataset $D = \{(x_i, y_i)\}_{i=1}^{N}$, where $y_i = f(x_i) + \epsilon_i$ and $\epsilon_i \sim \mathcal{N}(0, \sigma_n^2)$ represents independent Gaussian noise with variance σ_n^2, the GP posterior predictive distribution at a new point x_* remains Gaussian:

$$p(f(x_*) \mid x_*, D) = \mathcal{N}\big(f(x_*) \mid \mu_{\mathrm{GP}}(x_*), \sigma^2_{\mathrm{GP}}(x_*)\big). \tag{3}$$

The predictive mean $\mu_{\mathrm{GP}}(x_*)$ provides an estimate of the objective function value, while the predictive variance $\sigma^2_{\mathrm{GP}}(x_*)$ quantifies the associated uncertainty. These two quantities play a central role in guiding the optimization process.[S15] GPs are particularly attractive in BO due to their flexibility in modeling complex nonlinear relationships and their ability to naturally provide well-calibrated uncertainty estimates.[S15]

The acquisition function guides the selection of the next evaluation point by quantifying the expected benefit of sampling the objective function at a given location in the parameter space. Among the many acquisition functions proposed in the literature, Expected Improvement (EI) is one of the most widely used.[S15] EI measures the expected improvement over the best objective function value observed so far, thereby favoring points that either have a high predicted performance or a high associated uncertainty. Let $y_{\min}$ denote the best (minimum) observed objective function value. The improvement is defined as $I(x) = \max(0, y_{\min} - Y)$, where $Y \sim \mathcal{N}(\mu(x), \sigma^2(x))$ is the GP predictive distribution at point x. The EI admits the following closed-form expression:

$$EI(x) = \begin{cases} (y_{\min} - \mu(x) - \xi)\, \Phi(Z) + \sigma(x)\, \phi(Z), & \text{if } \sigma(x) > 0, \\ 0, & \text{if } \sigma(x) = 0, \end{cases} \tag{4}$$

where $Z = \frac{y_{\min} - \mu(x) - \xi}{\sigma(x)}$ and $\xi \geq 0$ is a parameter controlling the exploration–exploitation trade-off.[S17] Here, $\Phi(\cdot)$ and $\phi(\cdot)$ denote the cumulative distribution function and probability

density function of the standard normal distribution, respectively. In practice, EI provides a robust and intuitive balance between exploration and exploitation.

Other commonly used acquisition functions include Probability of Improvement (PI), Upper or Lower Confidence Bound (U/LCB), Thompson Sampling (TS) or probability matching, and entropy-based methods.[S15,S18]

The performance of BO depends critically on problem formulation, including the selection of relevant input variables (features or descriptors), their feasible ranges, and the numerical representation suitable for model learning.[S15] In materials science and chemistry, this often requires transforming molecular structures, compositions, or processing conditions into appropriate numerical descriptors.[S15] Poor feature representation may degrade surrogate model accuracy and optimization efficiency, highlighting the importance of thoughtful problem encoding.

Rietveld refinements

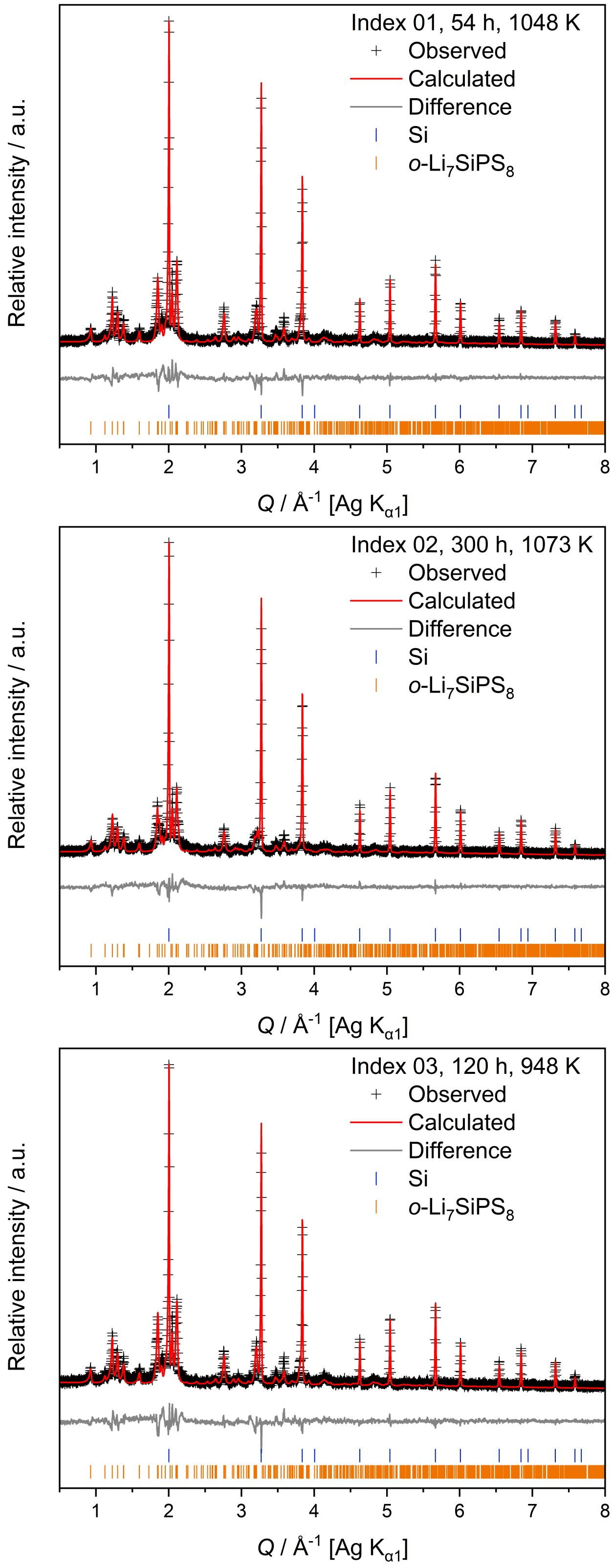

Figure S1: Rietveld refinement plots of samples 01-03. Shown are the observed data points (black crosses), the calculated Rietveld fit (red), the difference plot (grey), and the reflection markers for Si, *t*-, and *o*-Li_7SiPS_8 (blue, light orange, and dark orange, respectively). The corresponding crystallographic data obtained from the refinements are shown in Table S2.

Table S2: Additional crystallographic information obtained from the quantitative Rietveld refinements of XRPD data of samples 01-03 using Si as an internal standard. The phase content of the internal standard has been omitted with the other phase contents scaled accordingly. The standard deviations are given in parentheses.

Samples index		01	02	03
Temperature program		54 h, 1048 K	300 h, 1073 K	120 h, 948 K
Phase / wt%	t-Li_7SiPS_8	0	0	0
Space group			$P4_2/nmc$ (No. 132)	
Lattice parameters / Å	a	-	-	-
	c	-	-	-
Volume / Å^3		-	-	-
R_{Bragg}		-	-	-
Phase / wt%	o-Li_7SiPS_8	89.2(9)	87.2(9)	98(1)
Space group			$Pnma$ (No. 62)	
Lattice parameters / Å	a	13.524(3)	13.445(2)	13.547(4)
	b	7.813(2)	7.905(2)	7.862(3)
	c	6.136(1)	6.130(1)	6.136(1)
Volume / Å^3		653.3(2)	351.5(2)	653.6(3)
R_{Bragg}		6.0278	5.9648	5.6568
Phase / wt%	amorphous	10.8(8)	12.8(9)	2(1)
Diffractometer	Stoe Stadi P, Ag $K_{\alpha 1}$ radiation, Debye-Scherrer geometry			
Refined Q region / Å^{-1}		0.392-10.590	0.392-10.590	0.392-10.590
R_p		10.447	9.657	10.833
R_{wp}		13.788	12.728	14.147
R_{exp}		10.483	10.315	11.124
GoF		1.315	1.234	1.272
Number of refined parameters		34	27	34
Number of refined background parameters		4	4	4

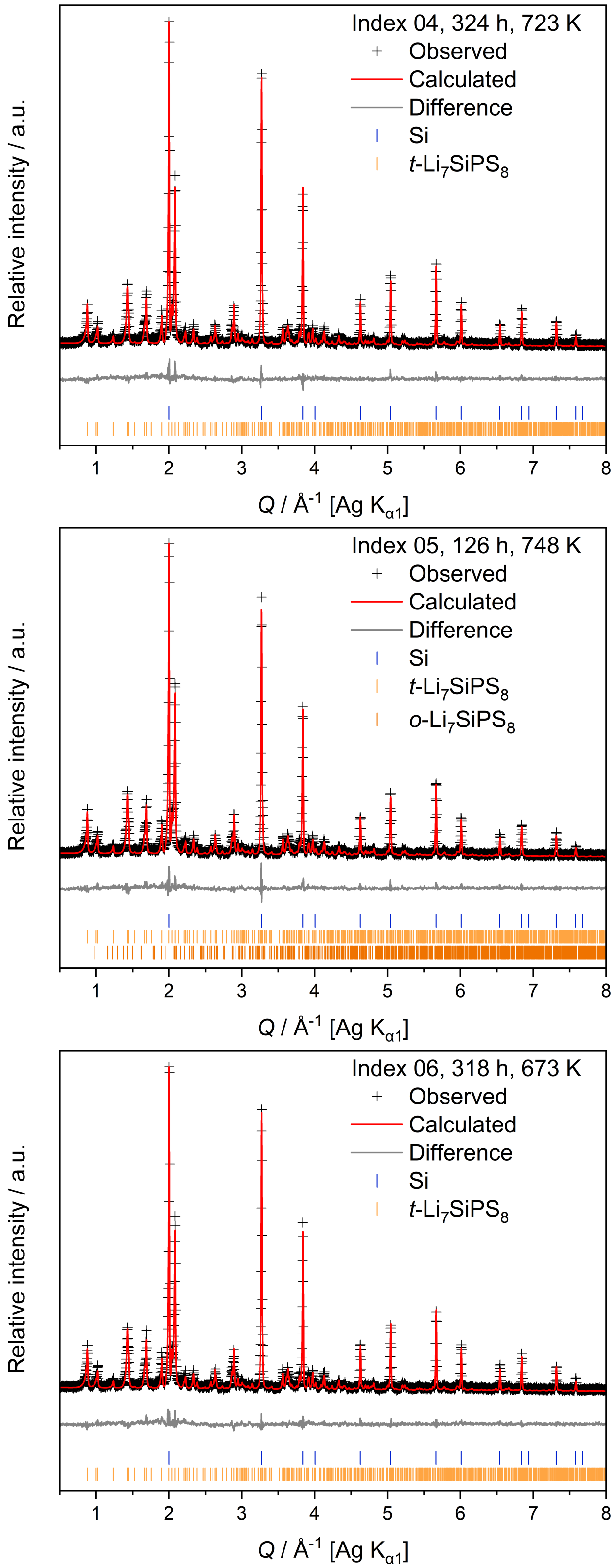

Figure S2: Rietveld refinement plots of samples 04-06. Shown are the observed data points (black crosses), the calculated Rietveld fit (red), the difference plot (grey), and the reflection markers for Si, *t*-, and *o*-Li_7SiPS_8 (blue, light orange, and dark orange, respectively). The corresponding crystallographic data obtained from the refinements are shown in Table S3.

Table S3: Additional crystallographic information obtained from the quantitative Rietveld refinements of XRPD data of samples 04-06 using Si as an internal standard. The phase content of the internal standard has been omitted with the other phase contents scaled accordingly. The standard deviations are given in parentheses.

Samples index		04	05	06
Temperature program		324 h, 723 K	126 h, 748 K	318 h, 673 K
Phase / wt%	t-Li_7SiPS_8	92.4(7)	92.2(7)	92.3(8)
Space group			$P4_2/nmc$ (No. 132)	
Lattice parameters / Å	a	8.6977(4)	8.6913(5)	8.7013(4)
	c	12.560(1)	12.555(1)	12.560(1)
Volume / $Å^3$		950.1(1)	948.4(1)	950.9(1)
R_{Bragg}		4.6581	4.0513	4.5701
Phase / wt%	o-Li_7SiPS_8	0	0	0
Space group			$Pnma$ (No. 62)	
Lattice parameters / Å	a	-	-	-
	b	-	-	-
	c	-	-	-
Volume / $Å^3$		-	-	-
R_{Bragg}		-	-	-
Phase / wt%	amorphous	7.6(7)	7.8(7)	7.7(8)
Diffractometer	Stoe Stadi P, Ag $K_{\alpha 1}$ radiation, Debye-Scherrer geometry			
Refined Q region / $Å^{-1}$		0.392-10.590	0.392-10.590	0.392-10.590
R_p		8.513	8.266	8.270
R_{wp}		11.172	10.802	10.877
R_{exp}		10.270	10.597	10.454
GoF		1.088	1.019	1.040
Number of refined parameters		27	38	31
Number of refined background parameters		4	4	4

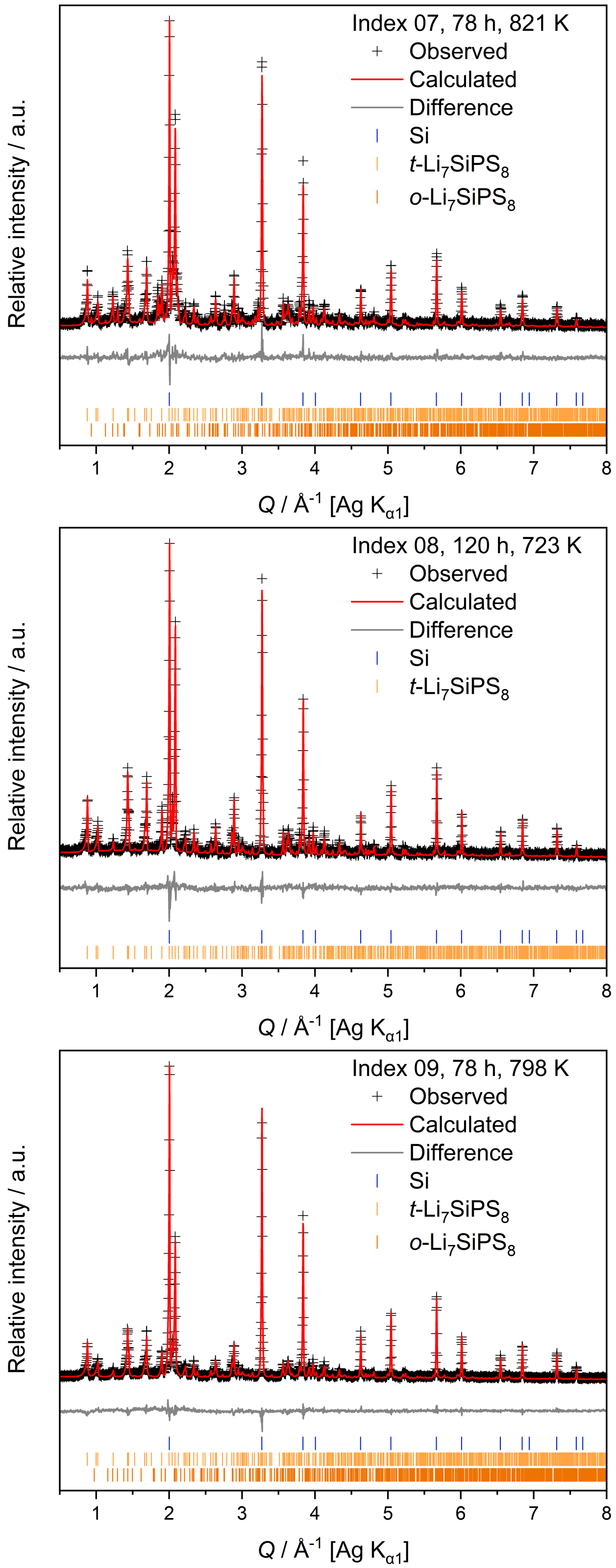

Figure S3: Rietveld refinement plots of samples 07-09. Shown are the observed data points (black crosses), the calculated Rietveld fit (red), the difference plot (grey), and the reflection markers for Si, *t*-, and *o*-Li_7SiPS_8 (blue, light orange, and dark orange, respectively). The corresponding crystallographic data obtained from the refinements are shown in Table S4.

Table S4: Additional crystallographic information obtained from the quantitative Rietveld refinements of XRPD data of samples 07-09 using Si as an internal standard. The phase content of the internal standard has been omitted with the other phase contents scaled accordingly. The standard deviations are given in parentheses.

Samples index		07	08	09
Temperature program		78 h, 548 K	120 h, 723 K	78 h, 798 K
Phase / wt%	t-Li_7SiPS_8	79.001(6)	95.30(1)	82.3(6)
Space group			$P4_2/nmc$ (No. 132)	
Lattice parameters / Å	a	8.7006(4)	8.6958(4)	8.7004(4)
	c	12.566(1)	12.55(1)	12.564(1)
Volume / Å^3		951.2(1)	949.5(1)	951.1(1)
R_{Bragg}		3.0722	5.7525	4.7984
Phase / wt%	o-Li_7SiPS_8	20.999(6)	0	0
Space group			$Pnma$ (No. 62)	
Lattice parameters / Å	a	13.453(2)	-	-
	b	7.908(1)	-	-
	c	6.124(1)	-	-
Volume / Å^3		651.6(2)	-	-
R_{Bragg}		5.1922	-	-
Phase / wt%	amorphous	0	4.70(1)	17.7(6)
Diffractometer	Stoe Stadi P, Ag $K_{\alpha1}$ radiation, Debye-Scherrer geometry			
Refined Q region / Å^{-1}		0.392-8.996	0.392-8.996	0.392-10.590
R_p		9.074	9.937	8.379
R_{wp}		12.003	12.882	10.986
R_{exp}		11.531	12.578	10.032
GoF		1.041	1.024	1.095
Number of refined parameters		50	34	38
Number of refined background parameters		10	10	4

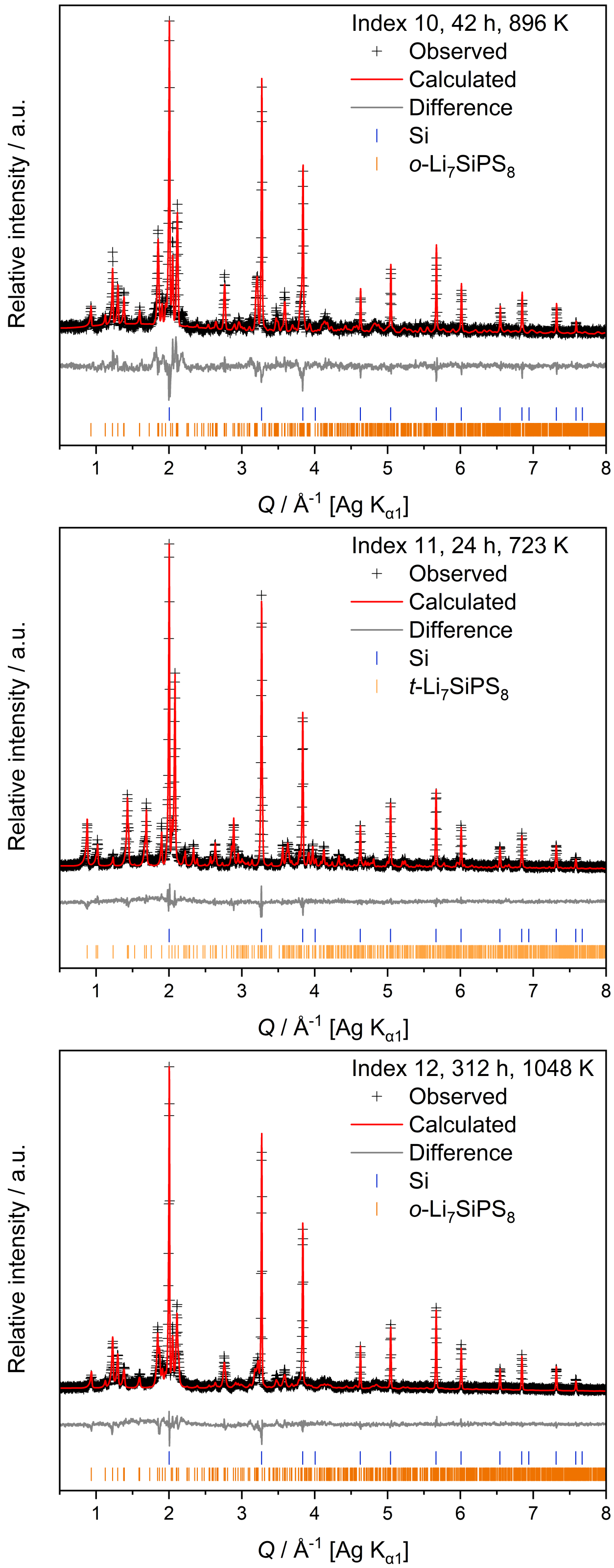

Figure S4: Rietveld refinement plots of samples 10-12. Shown are the observed data points (black crosses), the calculated Rietveld fit (red), the difference plot (grey), and the reflection markers for Si, *t*-, and *o*-Li_7SiPS_8 (blue, light orange, and dark orange, respectively). The corresponding crystallographic data obtained from the refinements are shown in Table S5.

Table S5: Additional crystallographic information obtained from the quantitative Rietveld refinements of XRPD data of samples 10-12 using Si as an internal standard. The phase content of the internal standard has been omitted with the other phase contents scaled accordingly. The standard deviations are given in parentheses.

Samples index		10	11	12
Temperature program		42 h, 623 K	24 h, 723 K	312 h, 1048 K
Phase / wt%	t-Li_7SiPS_8	0	98.9(8)	0
Space group			$P4_2/nmc$ (No. 132)	
Lattice parameters / Å	a	-	8.6996(4)	-
	c	-	12.560(1)	-
Volume / $Å^3$		-	950.6(1)	-
R_{Bragg}		-	4.5787	-
Phase / wt%	o-Li_7SiPS_8	68.52(2)	0	97(1)
Space group			$Pnma$ (No. 62)	
Lattice parameters / Å	a	13.522(2)	-	13.443(2)
	b	7.870(1)	-	7.907(1)
	c	6.1306(9)	-	6.1303(9)
Volume / $Å^3$		652.4(1)	-	651.7(2)
R_{Bragg}		10.469	-	5.5279
Phase / wt%	amorphous	31.48(2)	1.1(8)	3(1)
Diffractometer	Stoe Stadi P, Ag $K_{\alpha 1}$ radiation, Debye-Scherrer geometry			
Refined Q region / $Å^{-1}$		0.392-8.996	0.392-10.590	0.392-10.590
R_p		13.449	8.413	9.449
R_{wp}		17.391	11.095	12.351
R_{exp}		13.706	9.728	9.992
GoF		1.269	1.140	1.236
Number of refined parameters		33	27	27
Number of refined background parameters		10	4	4

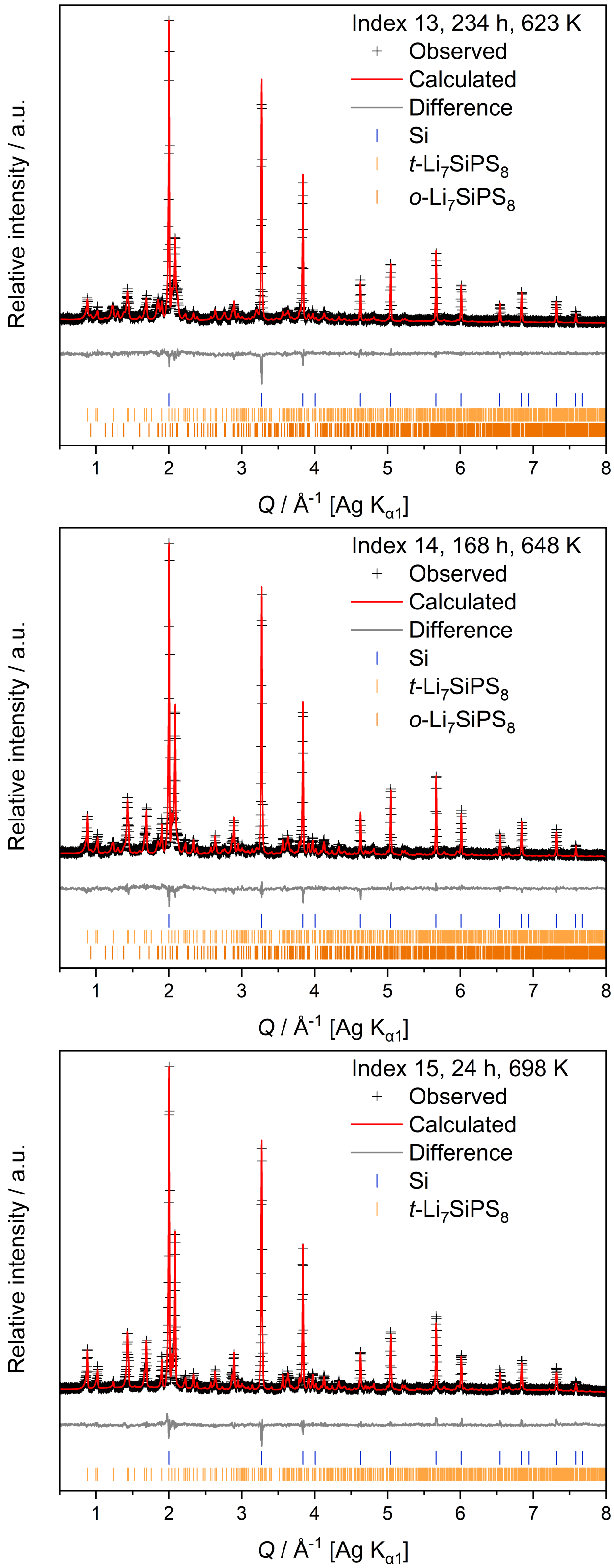

Figure S5: Rietveld refinement plots of samples 13-15. Shown are the observed data points (black crosses), the calculated Rietveld fit (red), the difference plot (grey), and the reflection markers for Si, *t*-, and *o*-Li_7SiPS_8 (blue, light orange, and dark orange, respectively). The corresponding crystallographic data obtained from the refinements are shown in Table S6.

Table S6: Additional crystallographic information obtained from the quantitative Rietveld refinements of XRPD data of samples 13-15 using Si as an internal standard. The phase content of the internal standard has been omitted with the other phase contents scaled accordingly. The standard deviations are given in parentheses.

Samples index		13	14	15
Temperature program		234 h, 623 K	168 h, 648 K	24 h, 698 K
Phase / wt%	t-Li_7SiPS_8	54.3(9)	76.8(8)	85(1)
Space group			$P4_2/nmc$ (No. 132)	
Lattice parameters / Å	a	8.6974(8)	8.7016(5)	8.6978(4)
	c	12.552(2)	12.561(1)	12.5588(9)
Volume / $Å^3$		949.5(2)	951.1(1)	950.1(1)
R_{Bragg}		4.4685	4.0515	2.9820
Phase / wt%	o-Li_7SiPS_8	36.0(9)	22.7(9)	0
Space group			$Pnma$ (No. 62)	
Lattice parameters / Å	a	13.56(1)	13.56(1)	-
	b	7.867(7)	7.87(1)	-
	c	6.125(3)	6.120(5)	-
Volume / $Å^3$		653.5(8)	653(1)	-
R_{Bragg}		4.2927	4.4021	-
Phase / wt%	amorphous	9.7(9)	0.4(10)	15(1)
Diffractometer	Stoe Stadi P, Ag $K_{\alpha 1}$ radiation, Debye-Scherrer geometry			
Refined Q region / $Å^{-1}$		0.392-10.590	0.392-10.590	0.392-8.996
R_p		8.306	7.788	7.609
R_{wp}		10.780	10.197	10.323
R_{exp}		10.508	9.955	8.958
GoF		1.026	1.024	1.152
Number of refined parameters		39	39	30
Number of refined background parameters		4	4	10

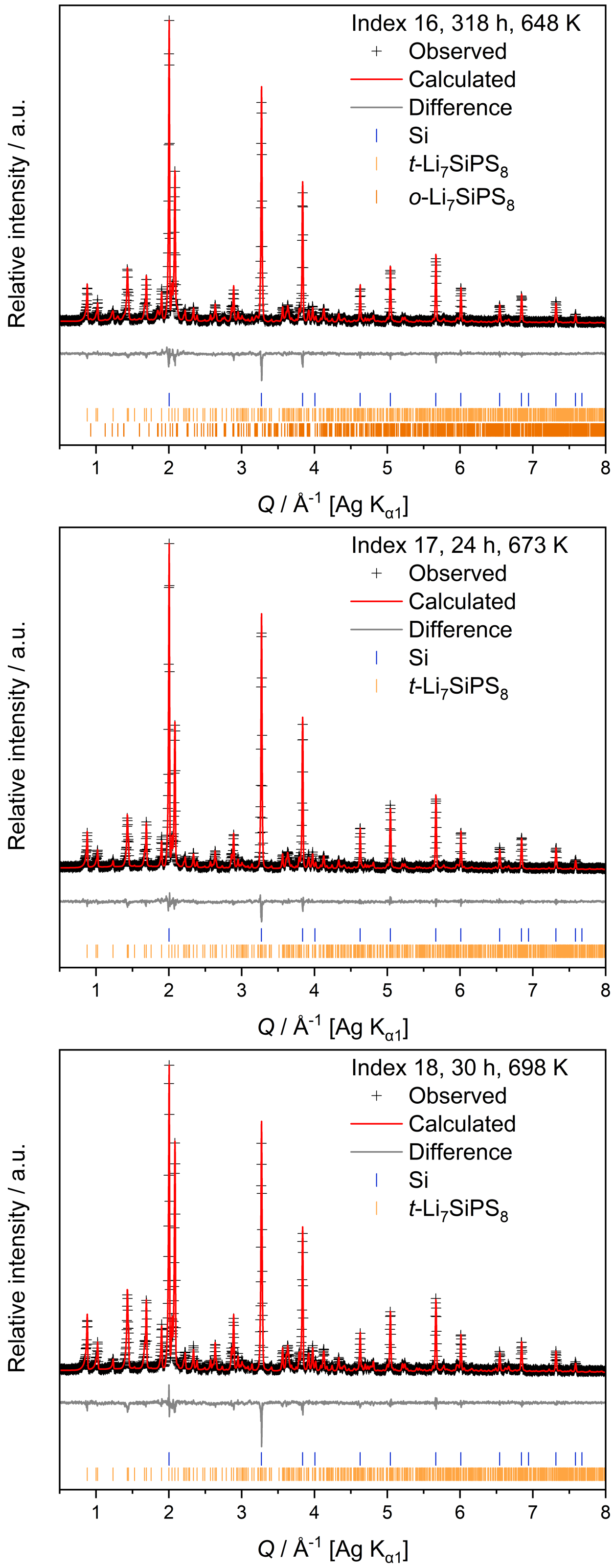

Figure S6: Rietveld refinement plots of samples 16-18. Shown are the observed data points (black crosses), the calculated Rietveld fit (red), the difference plot (grey), and the reflection markers for Si, *t*-, and *o*-Li_7SiPS_8 (blue, light orange, and dark orange, respectively). The corresponding crystallographic data obtained from the refinements are shown in Table S7.

Table S7: Additional crystallographic information obtained from the quantitative Rietveld refinements of XRPD data of samples 16-18 using Si as an internal standard. The phase content of the internal standard has been omitted with the other phase contents scaled accordingly. The standard deviations are given in parentheses.

Samples index		16	17	18
Temperature program		318 h, 648 K	24 h, 673 K	30 h, 698 K
Phase / wt%	t-Li_7SiPS_8	79.0(9)	93(1)	100
Space group			$P4_2/nmc$ (No. 132)	
Lattice parameters / Å	a	8.6965(3)	8.6983(3)	8.6974(3)
	c	12.5528(8)	12.557(8)	12.5554(7)
Volume / $Å^3$		949.36(9)	950.05(9)	949.75(8)
R_{Bragg}		2.7393	2.6816	2.9005
Phase / wt%	o-Li_7SiPS_8	17.4(6)	0	0
Space group			$Pnma$ (No. 62)	
Lattice parameters / Å	a	13.554(9)	-	-
	b	7.871(6)	-	-
	c	6.120(3)	-	-
Volume / $Å^3$		652.9(8)	-	-
R_{Bragg}		3.6345	-	-
Phase / wt%	amorphous	3.6(12)	7(1)	0
Diffractometer	Stoe Stadi P, Ag $K_{\alpha 1}$ radiation, Debye-Scherrer geometry			
Refined Q region / $Å^{-1}$		0.392-8.996	0.392-8.996	0.392-8.996
R_p		6.695	7.109	6.654
R_{wp}		8.645	9.417	8.569
R_{exp}		8.519	9.415	8.615
GoF		1.015	1.000	0.995
Number of refined parameters		38	30	40
Number of refined background parameters		10	10	10

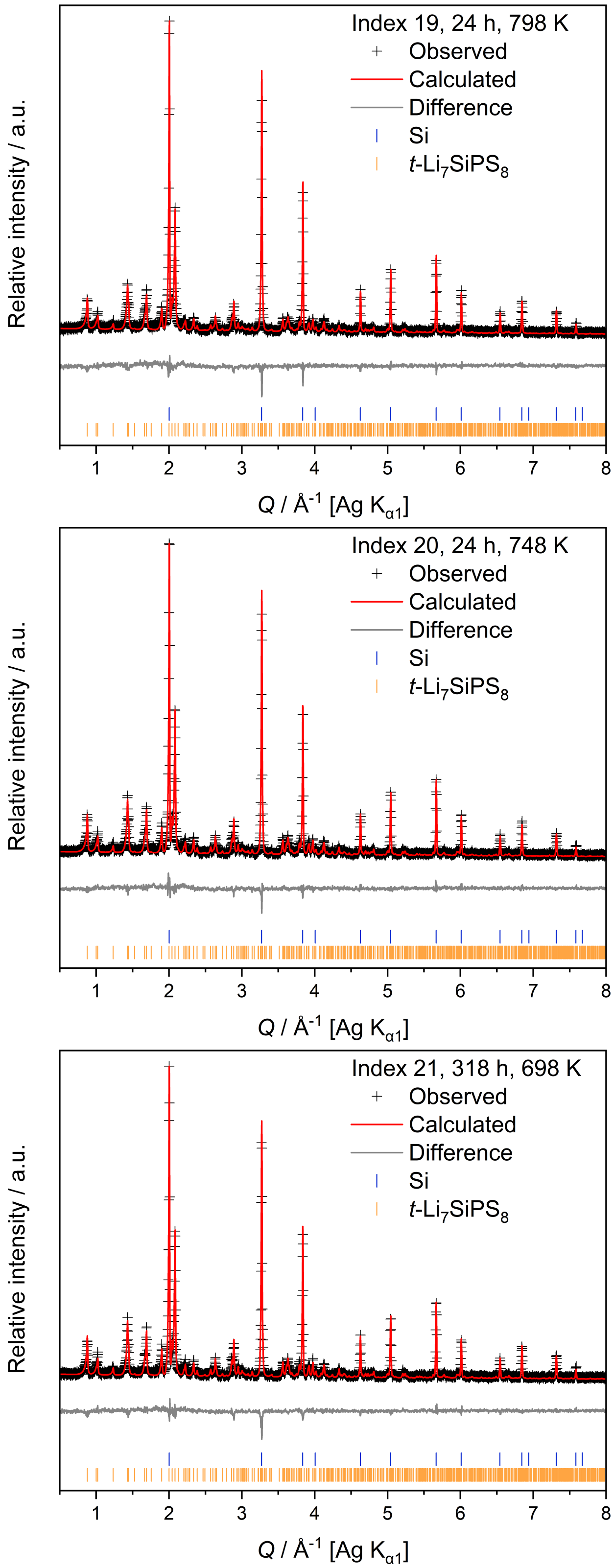

Figure S7: Rietveld refinement plots of samples 19-21. Shown are the observed data points (black crosses), the calculated Rietveld fit (red), the difference plot (grey), and the reflection markers for Si, *t*-, and *o*-Li_7SiPS_8 (blue, light orange, and dark orange, respectively). The corresponding crystallographic data obtained from the refinements are shown in Table S8.

Table S8: Additional crystallographic information obtained from the quantitative Rietveld refinements of XRPD data of samples 19-21 using Si as an internal standard. The phase content of the internal standard has been omitted with the other phase contents scaled accordingly. The standard deviations are given in parentheses.

Samples index		19	20	21
Temperature program		24 h, 798 K	24 h, 748 K	318 h, 698 K
Phase / wt%	t-Li_7SiPS_8	84.2(9)	89.4(8)	88.5(8)
Space group			$P4_2/nmc$ (No. 132)	
Lattice parameters / Å	a	8.6984(8)	8.6981(6)	8.6963(6)
	c	12.563(2)	12.560(1)	12.559(1)
Volume / $Å^3$		950.5(2)	950.2(2)	949.8(2)
R_{Bragg}		4.8633	3.7278	4.3893
Phase / wt%	o-Li_7SiPS_8	0	0	0
Space group			$Pnma$ (No. 62)	
Lattice parameters / Å	a	-	-	-
	b	-	-	-
	c	-	-	-
Volume / $Å^3$		-	-	-
R_{Bragg}		-	-	-
Phase / wt%	amorphous	15.8(9)	10.6(8)	11.5(8)
Diffractometer	Stoe Stadi P, Ag $K_{\alpha1}$ radiation, Debye-Scherrer geometry			
Refined Q region / $Å^{-1}$		0.392-10.590	0.392-10.590	0.392-10.590
R_p		9.352	9.109	8.875
R_{wp}		12.092	11.969	11.583
R_{exp}		12.106	12.054	11.435
GoF		0.999	0.993	1.013
Number of refined parameters		27	27	27
Number of refined background parameters		4	4	4

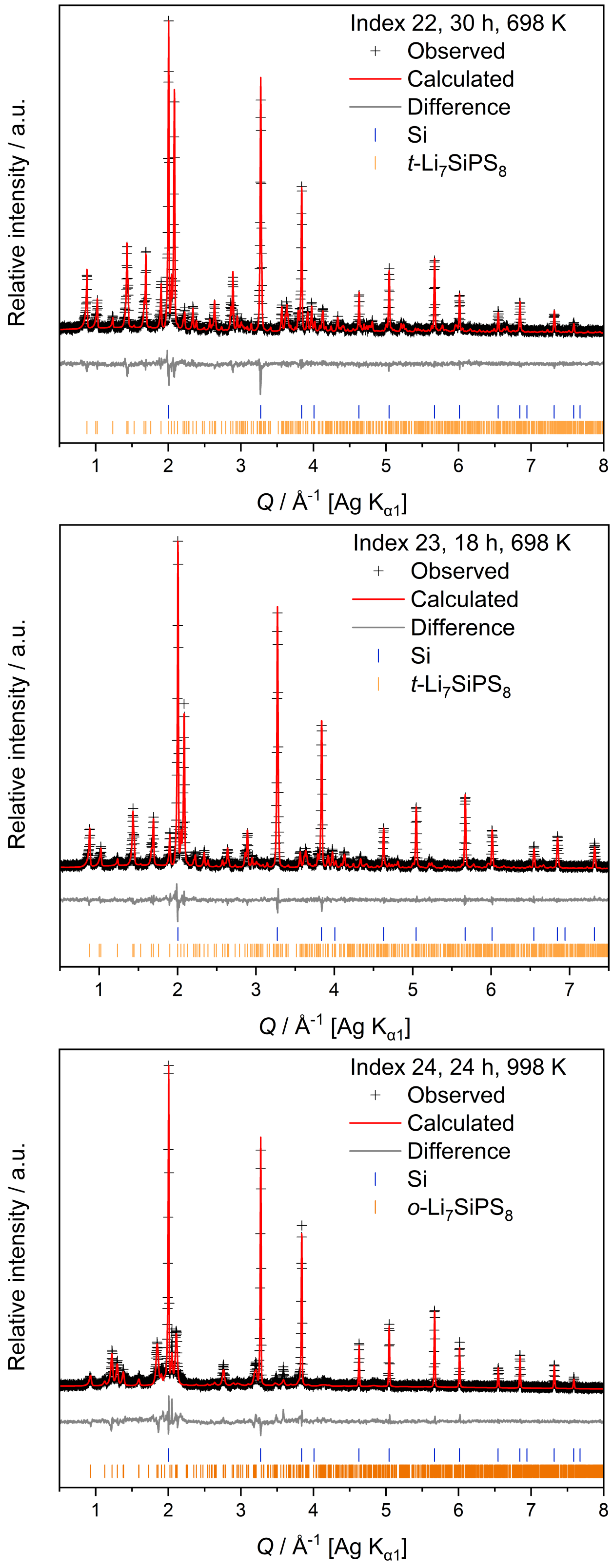

Figure S8: Rietveld refinement plots of samples 22-24. Shown are the observed data points (black crosses), the calculated Rietveld fit (red), the difference plot (grey), and the reflection markers for Si, *t*-, and *o*-Li_7SiPS_8 (blue, light orange, and dark orange, respectively). The corresponding crystallographic data obtained from the refinements are shown in Table S9.

Table S9: Additional crystallographic information obtained from the quantitative Rietveld refinements of XRPD data of samples 22-24 using Si as an internal standard. The phase content of the internal standard has been omitted with the other phase contents scaled accordingly. The standard deviations are given in parentheses.

Samples index		22	23	24
Temperature program		60 h, 689 K	18 h, 689 K	24 h, 998 K
Phase / wt%	t-Li_7SiPS_8	100	75(1)	0
Space group			$P4_2/nmc$ (No. 132)	
Lattice parameters / Å	a	8.6951(3)	8.6957(4)	-
	c	12.5546(8)	12.555(1)	-
Volume / $Å^3$		949.2(1)	949.4	-
R_{Bragg}		3.1514	2.7786	-
Phase / wt%	o-Li_7SiPS_8	0	0	96.8(9)
Space group			$Pnma$ (No. 62)	
Lattice parameters / Å	a	-	-	13.516(4)
	b	-	-	7.889(2)
	c	-	-	6.131(1)
Volume / $Å^3$		-	-	653.7(3)
R_{Bragg}		-	-	7.0355
Phase / wt%	amorphous	0	25(1)	3.2(9)
Diffractometer	Stoe Stadi P, Ag $K_{\alpha 1}$ radiation, Debye-Scherrer geometry			
Refined Q region / $Å^{-1}$		0.392-8.996	0.392-7.531	0.392-10.592
R_p		8.225	7.755	10.722
R_{wp}		10.727	10.403	14.180
R_{exp}		10.983	10.630	11.153
GoF		0.977	0.979	1.271
Number of refined parameters		37	37	21
Number of refined background parameters		10	10	4

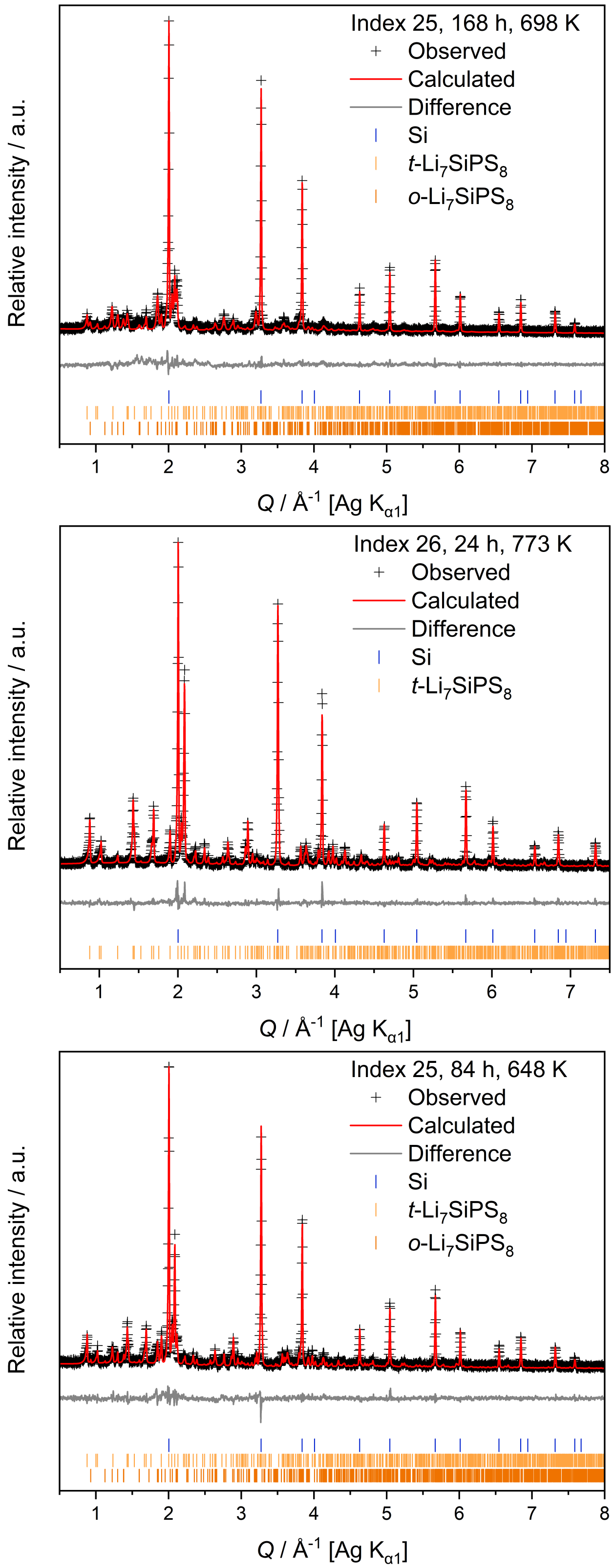

Figure S9: Rietveld refinement plots of samples 25-27. Shown are the observed data points (black crosses), the calculated Rietveld fit (red), the difference plot (grey), and the reflection markers for Si, *t*-, and *o*-Li_7SiPS_8 (blue, light orange, and dark orange, respectively). The corresponding crystallographic data obtained from the refinements are shown in Table S10.

Table S10: Additional crystallographic information obtained from the quantitative Rietveld refinements of XRPD data of samples 25-27 using Si as an internal standard. The phase content of the internal standard has been omitted with the other phase contents scaled accordingly. The standard deviations are given in parentheses.

Samples index		25	26	27
Temperature program		168 h, 698 K	24 h, 773 K	84 h, 648 K
Phase / wt%	t-Li_7SiPS_8	30.3(8)	91.3(9)	72(1)
Space group			$P4_2/nmc$ (No. 132)	
Lattice parameters / Å	a	8.6885(2)	8.6945(3)	8.6941(7)
	c	12.559(4)	12.5572(8)	12.546(2)
Volume / $Å^3$		948.0(5)	949.25(9)	948.3(2)
R_{Bragg}		5.7247	3.4480	4.4264
Phase / wt%	o-Li_7SiPS_8	53.3(8)	0	24(1)
Space group			$Pnma$ (No. 62)	
Lattice parameters / Å	a	13.627(5)	-	13.522(2)
	b	7.822(3)	-	7.874(1)
	c	6.131(2)	-	6.122(1)
Volume / $Å^3$		653.5(4)	-	651.8(2)
R_{Bragg}		6.1626	-	4.7937
Phase / wt%	amorphous	16.4(9)	8.7(9)	3(2)
Diffractometer	Stoe Stadi P, Ag $K_{\alpha1}$ radiation, Debye-Scherrer geometry			
Refined Q region / $Å^{-1}$		0.392-10.591	0.392-9.535	0.392-9.535
R_p		10.333	8.322	10.887
R_{wp}		13.763	11.806	14.337
R_{exp}		11.827	11.209	14.002
GoF		1.164	0.989	1.024
Number of refined parameters		33	37	50
Number of refined background parameters		4	10	10

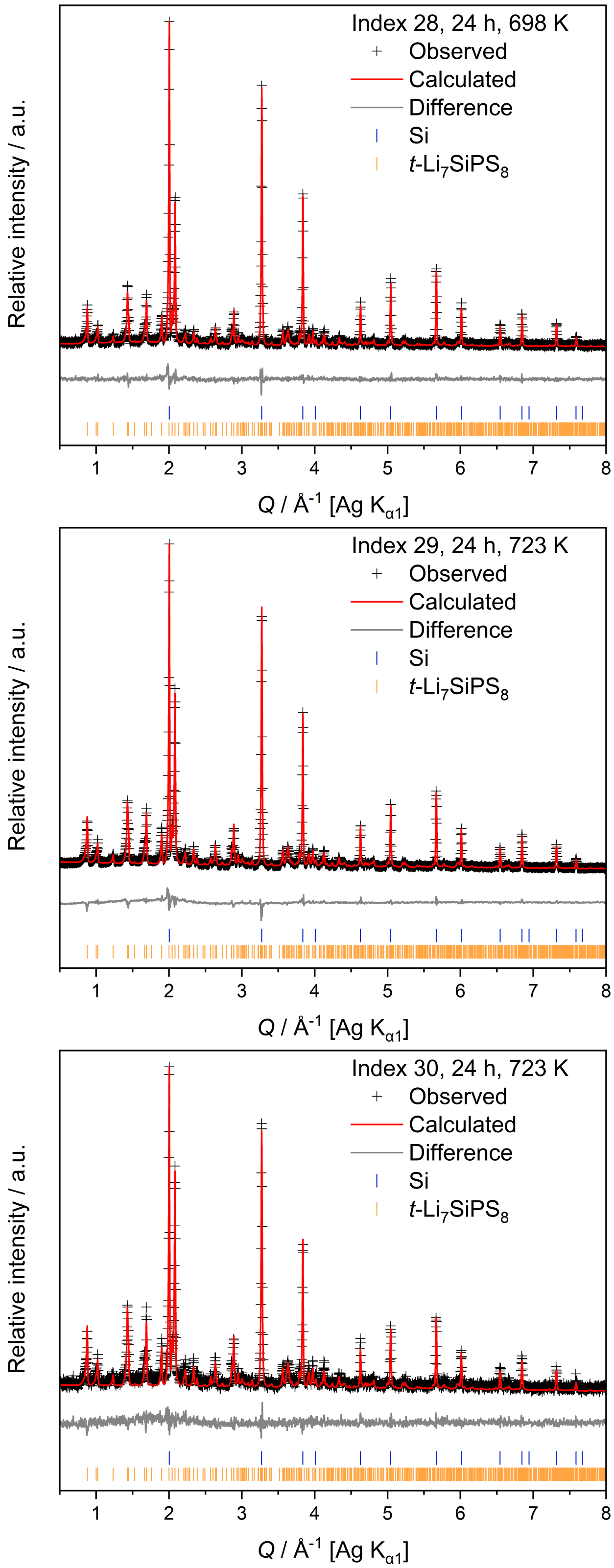

Figure S10: Rietveld refinement plots of samples 28-30. Shown are the observed data points (black crosses), the calculated Rietveld fit (red), the difference plot (grey), and the reflection markers for Si, *t*-, and *o*-Li_7SiPS_8 (blue, light orange, and dark orange, respectively). The corresponding crystallographic data obtained from the refinements are shown in Table S11.

Table S11: Additional crystallographic information obtained from the quantitative Rietveld refinements of XRPD data of samples 28-30 using Si as an internal standard. The phase content of the internal standard has been omitted with the other phase contents scaled accordingly. The standard deviations are given in parentheses.

Samples index		28	29	30
Temperature program		24 h, 698 K	24 h, 723 K	24 h, 723 K
Phase / wt%	t-Li_7SiPS_8	86(1)	88.0(5)	100
Space group			$P4_2/nmc$ (No. 132)	
Lattice parameters / Å	a	8.6941(7)	8.6965(4)	8.6957(8)
	c	12.546(2)	12.5563(8)	12.556(2)
Volume / $Å^3$		948.3(2)	949.6(1)	949.4(2)
R_{Bragg}		4.4264	4.2586	5.0650
Phase / wt%	o-Li_7SiPS_8	0	0	0
Space group			$Pnma$ (No. 62)	
Lattice parameters / Å	a	-	-	-
	b	-	-	-
	c	-	-	-
Volume / $Å^3$		-	-	-
R_{Bragg}		-	-	-
Phase / wt%	amorphous	14(1)	12.0(5)	0
Diffractometer	Stoe Stadi P, Ag $K_{\alpha1}$ radiation, Debye-Scherrer geometry			
Refined Q region / $Å^{-1}$		0.392-9.535	0.392-10.591	0.392-10.591
R_p		8.980	6.019	13.756
R_{wp}		11.971	7.965	18.347
R_{exp}		12.295	3.564	18.913
GoF		0.974	2.235	0.970
Number of refined parameters		33	24	24
Number of refined background parameters		10	4	4

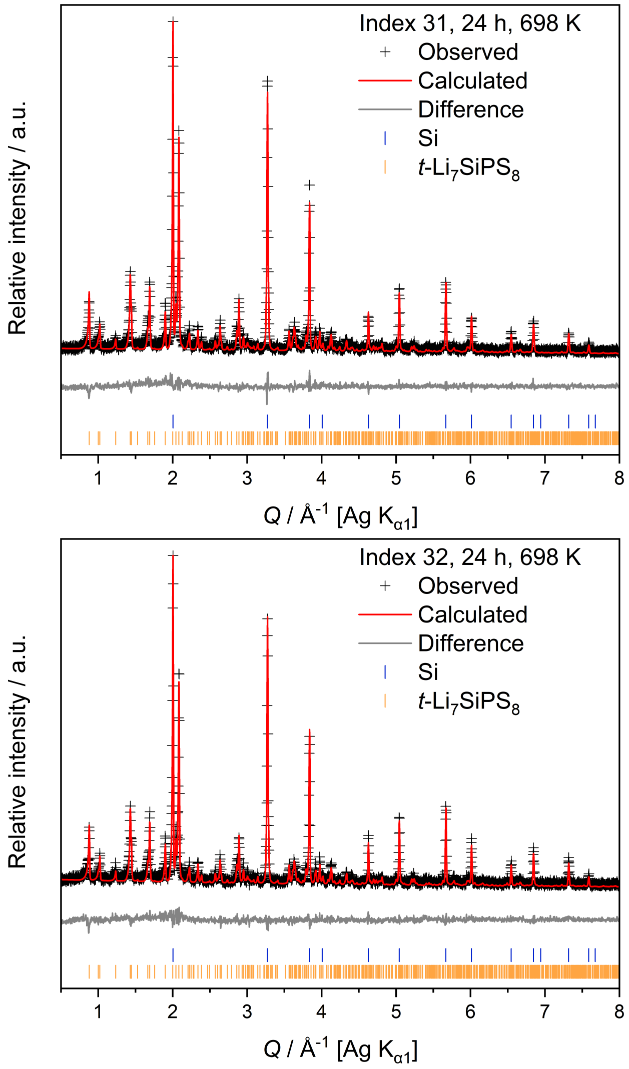

Figure S11: Rietveld refinement plots of samples 31 and 32. Shown are the observed data points (black crosses), the calculated Rietveld fit (red), the difference plot (grey), and the reflection markers for Si, *t*-, and *o*-Li_7SiPS_8 (blue, light orange, and dark orange, respectively). The corresponding crystallographic data obtained from the refinements are shown in Table S12.

Table S12: Additional crystallographic information obtained from the quantitative Rietveld refinements of XRPD data of samples 31 and 32 using Si as an internal standard. The phase content of the internal standard has been omitted with the other phase contents scaled accordingly. The standard deviations are given in parentheses.

Samples index		31	32
Temperature program		24 h, 698 K	24 h, 698 K
Phase / wt%	*t*-Li_7SiPS_8	97(1)	91(1)
Space group			$P4_2/nmc$ (No. 132)
Lattice parameters / Å	*a*	8.6941(5)	8.6938(6)
	c	12.553(1)	12.555(1)
Volume / $Å^3$		948.8(2)	948.9(2)
R_{Bragg}		5.0078	4.9587
Phase / wt%	*o*-Li_7SiPS_8	0	0
Space group			*Pnma* (No. 62)
Lattice parameters / Å	*a*	-	-
	b	-	-
	c	-	-
Volume / $Å^3$		-	-
R_{Bragg}		-	-
Phase / wt%	amorphous	3(1)	9(1)
Diffractometer	Stoe Stadi P, Ag $K_{\alpha1}$ radiation, Debye-Scherrer geometry		
Refined Q region / $Å^{-1}$		0.392-10.591	0.392-10.591
R_p		11.068	11.310
R_{wp}		14.701	15.203
R_{exp}		14.194	15.954
GoF		1.036	0.953
Number of refined parameters		24	24
Number of refined background parameters		4	4

Electrochemical Impedance Spectroscopy

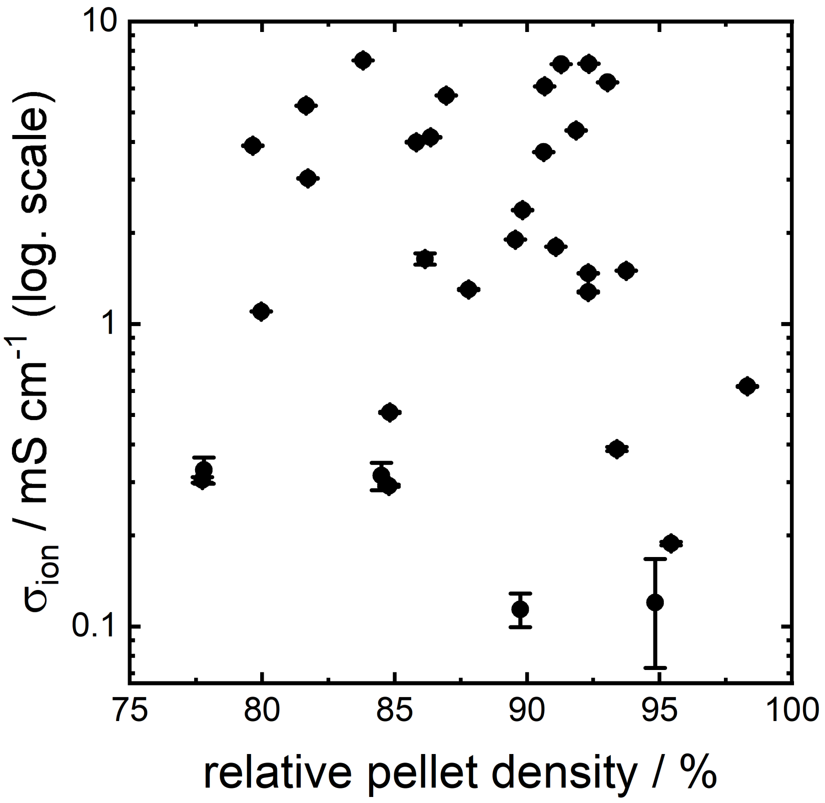

Figure S12: Logarithmic plot of the ionic conductivities plotted against the pellet densities.

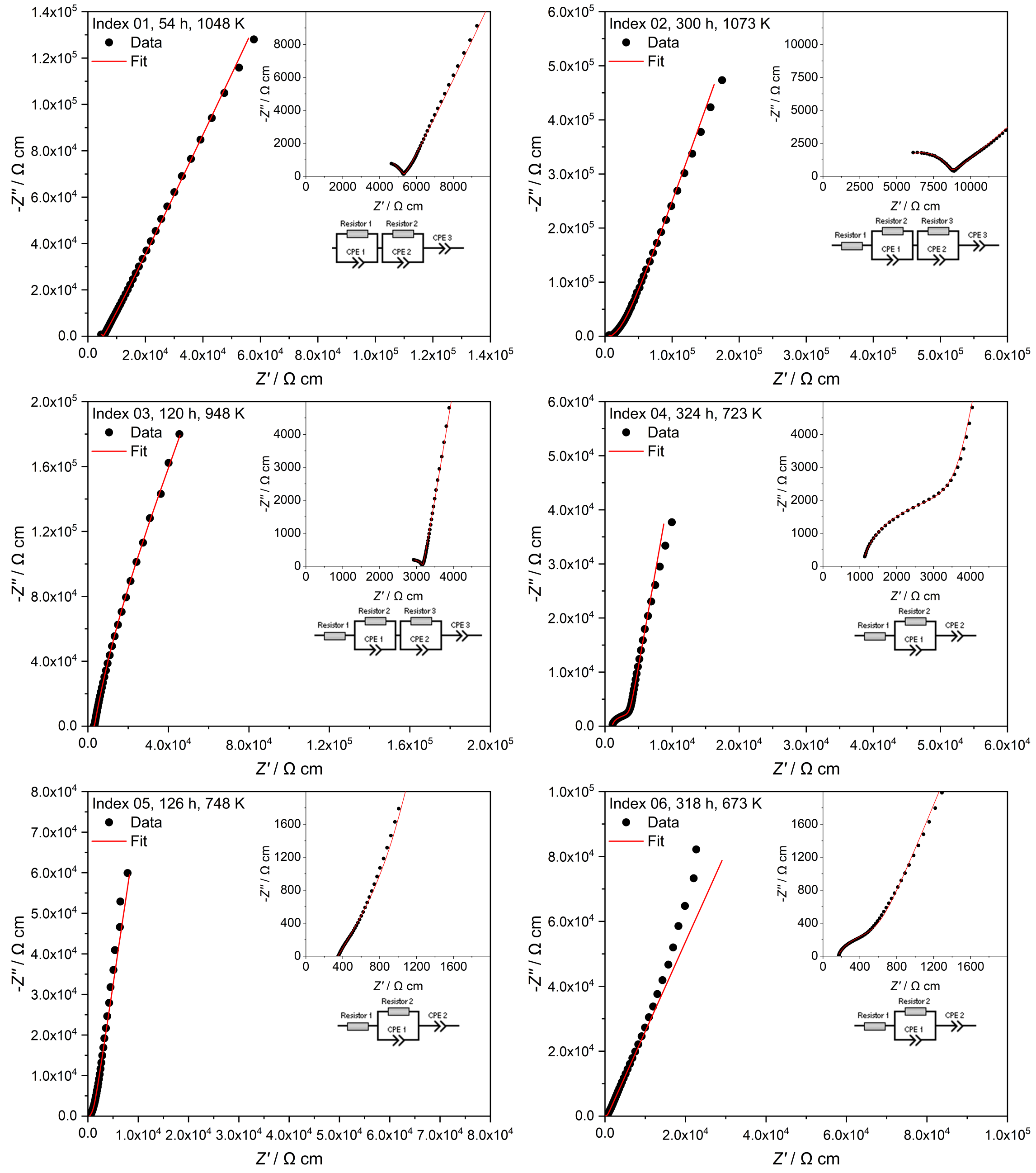

Figure S13: Nyquist plots of experimental EIS data (black circles) and equivalent circuit fits (red line) for samples 01-06. The inset shows an enlarged portion of the high-frequency region. The equivalent circuit model used for fitting is indicated in each plot.

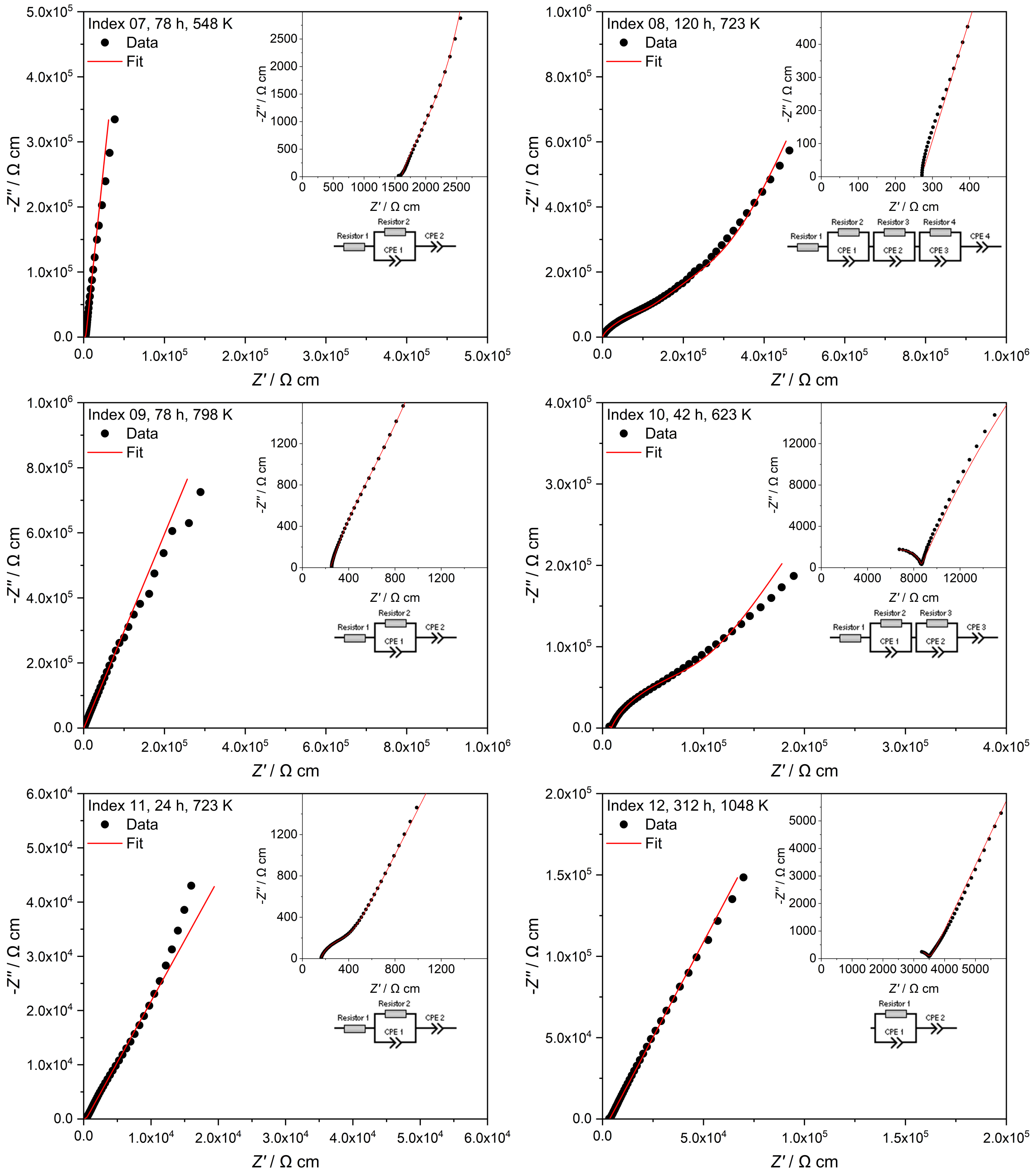

Figure S14: Nyquist plots of experimental EIS data (black circles) and equivalent circuit fits (red line) for samples 07-12. The inset shows an enlarged portion of the high-frequency region. The equivalent circuit model used for fitting is indicated in each plot.

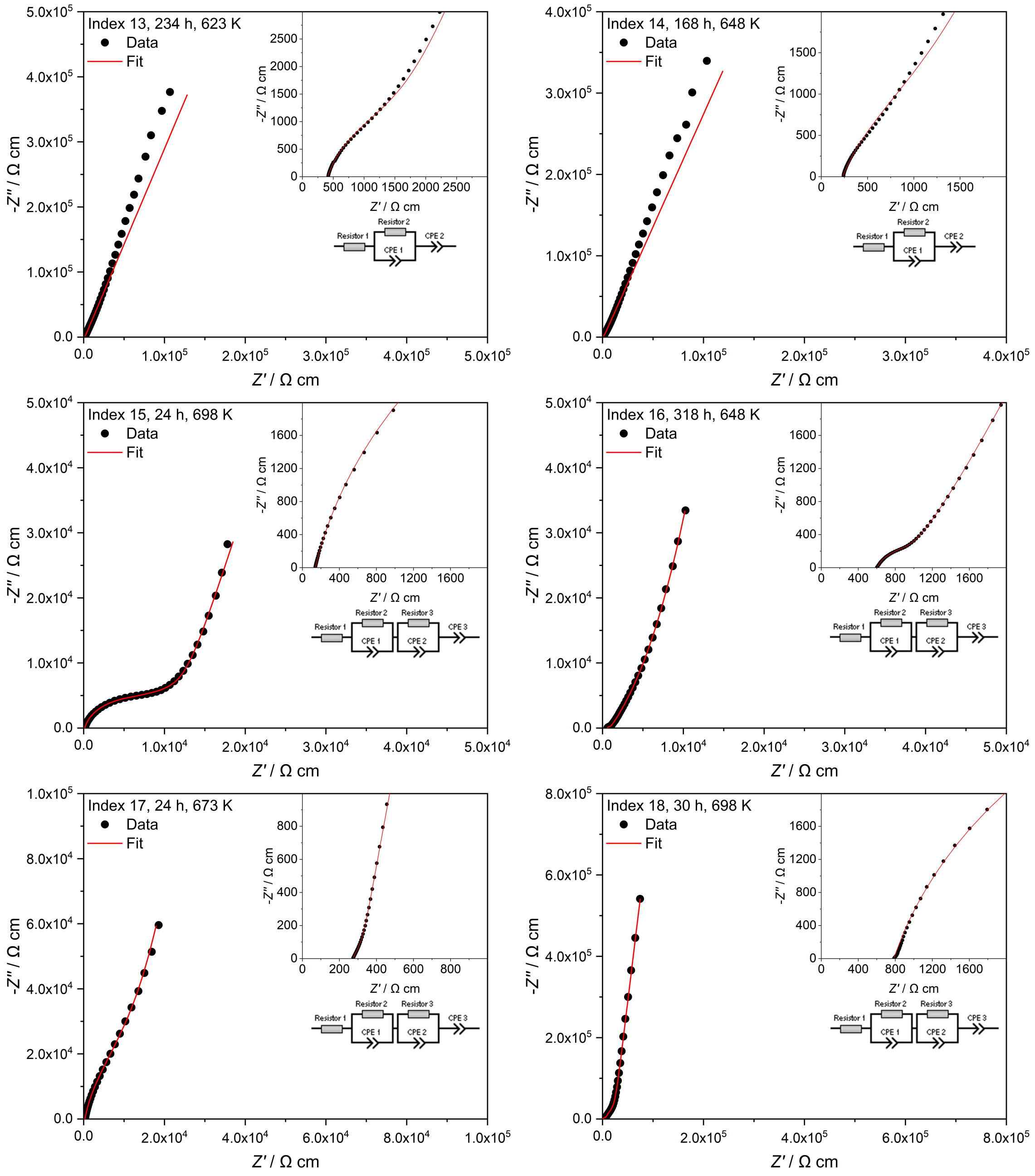

Figure S15: Nyquist plots of experimental EIS data (black circles) and equivalent circuit fits (red line) for samples 13-18. The inset shows an enlarged portion of the high-frequency region. The equivalent circuit model used for fitting is indicated in each plot.

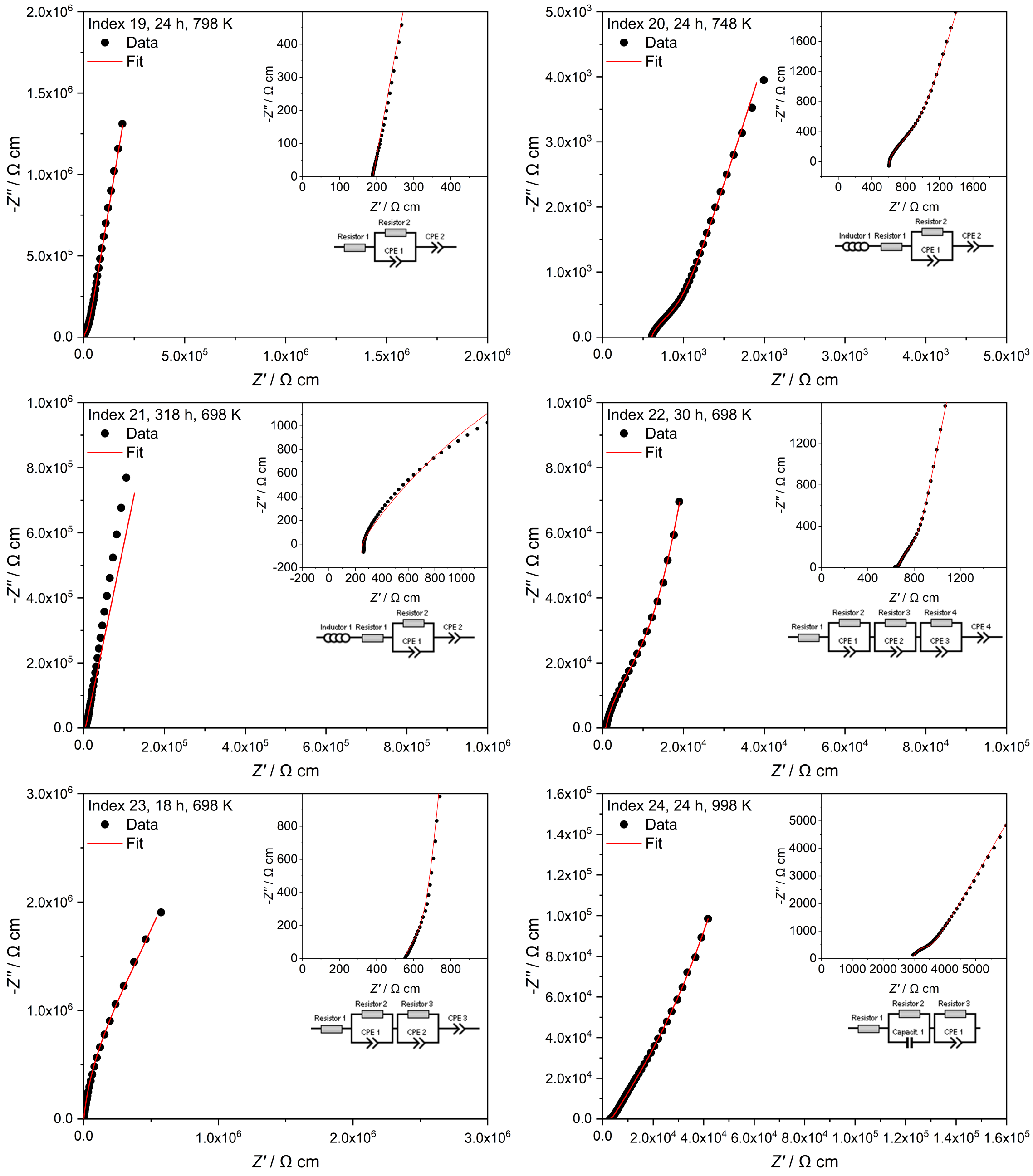

Figure S16: Nyquist plots of experimental EIS data (black circles) and equivalent circuit fits (red line) for samples 19-24. The inset shows an enlarged portion of the high-frequency region. The equivalent circuit model used for fitting is indicated in each plot.

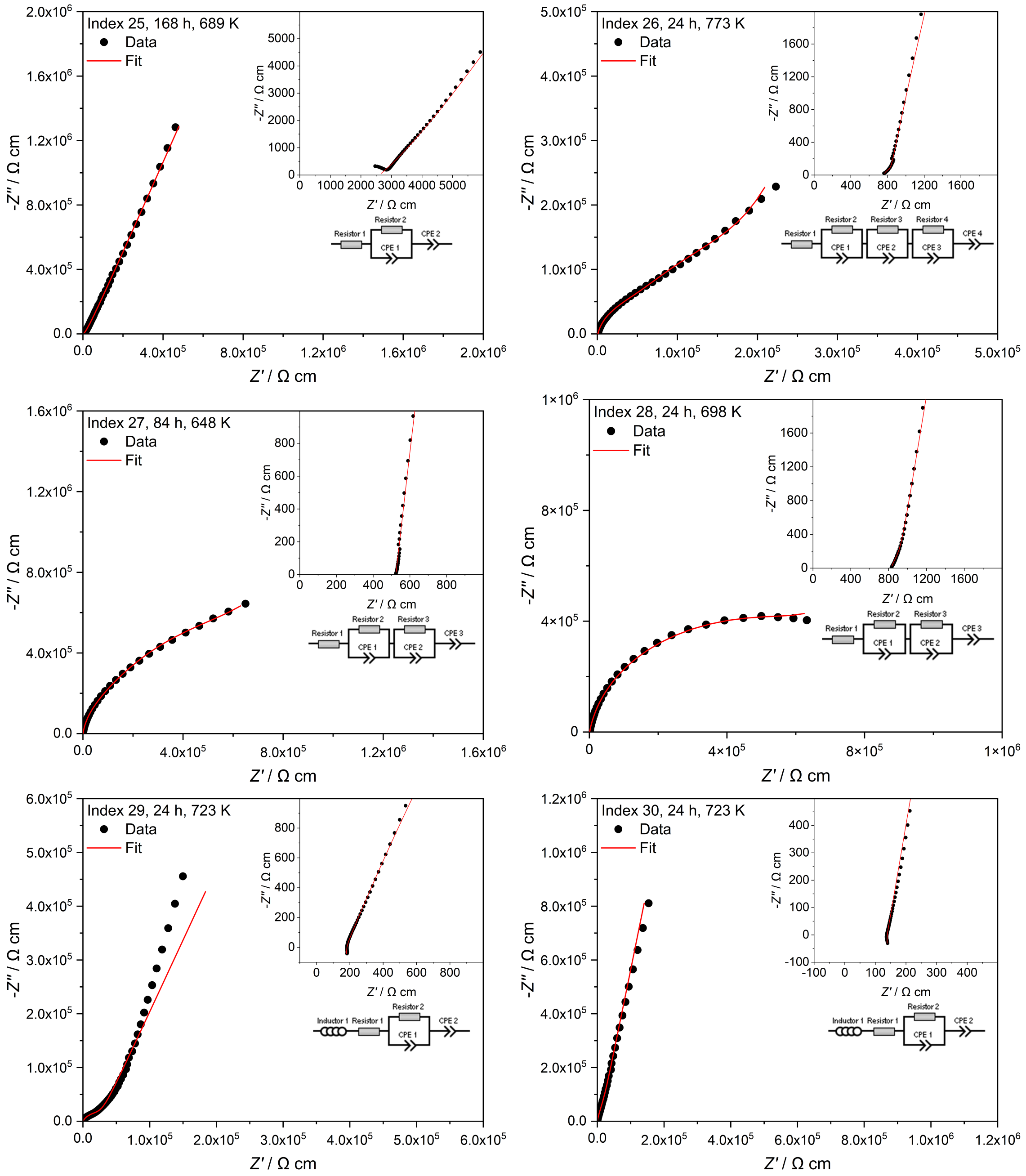

Figure S17: Nyquist plots of experimental EIS data (black circles) and equivalent circuit fits (red line) for samples 25-30. The inset shows an enlarged portion of the high-frequency region. The equivalent circuit model used for fitting is indicated in each plot.

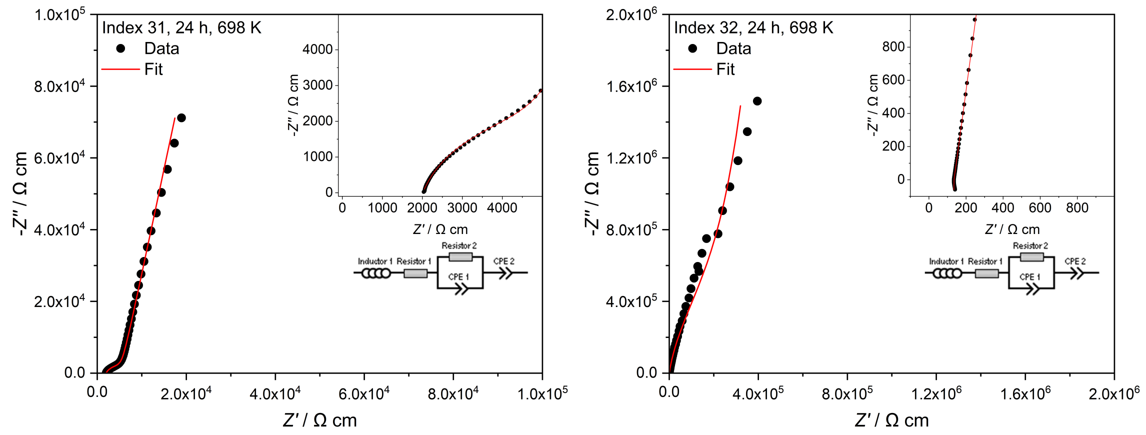

Figure S18: Nyquist plots of experimental EIS data (black circles) and equivalent circuit fits (red line) for samples 31 and 32. The inset shows an enlarged portion of the high-frequency region. The equivalent circuit model used for fitting is indicated in each plot.

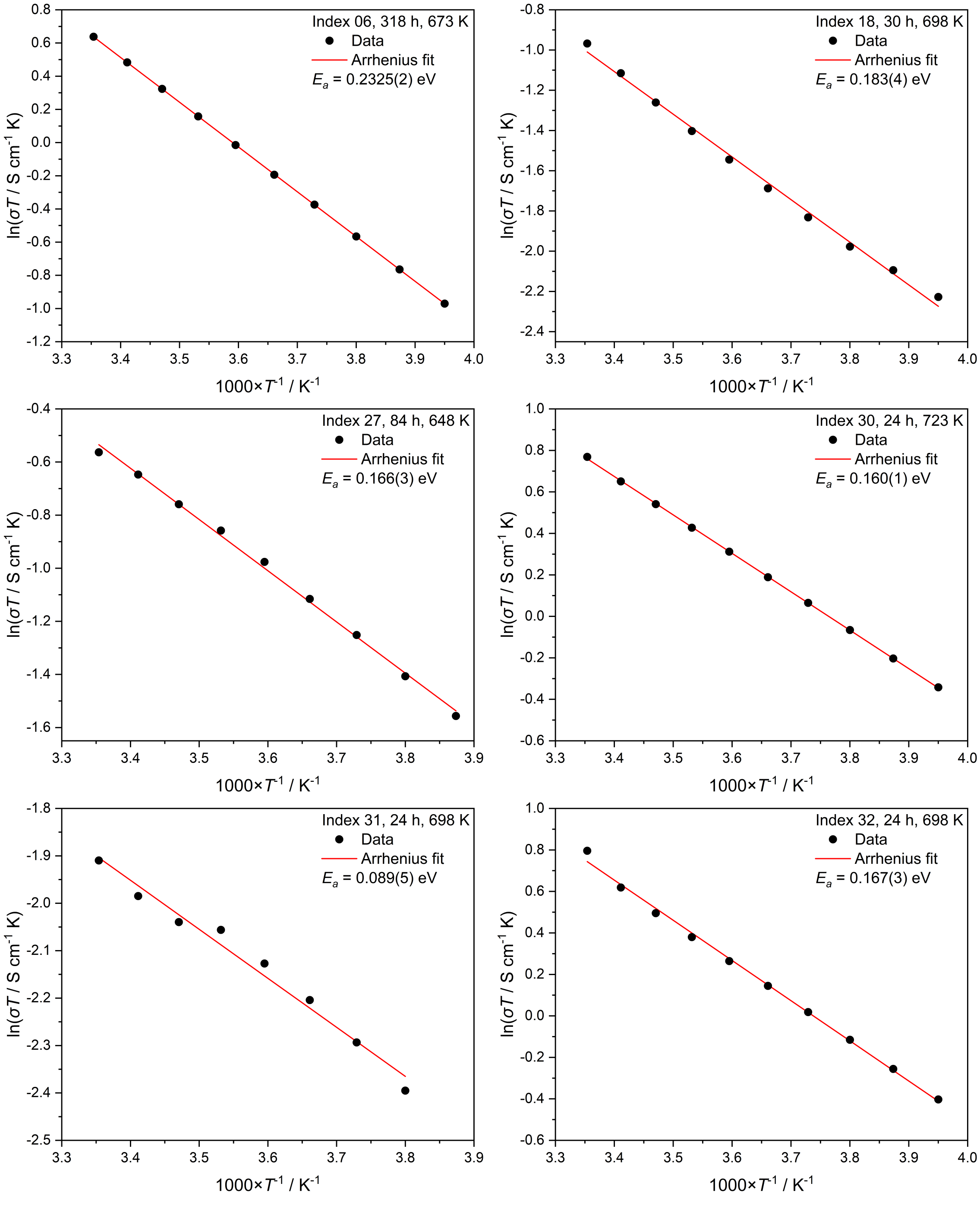

Figure S19: Arrhenius plots of samples with the indices 06, 18, 27, 30, 31, and 32, which were selected for further analysis. The graphs show temperature-dependent conductivity data (black circles) and the linear Arrhenius fit (red line)

Scanning electron microscopy & energy-dispersive X-ray spectroscopy

In the following figures, scanning electron microscopy (SEM) images as well as energy-dispersive X-ray (EDX) spectra of the different *tetra*-Li_7SiPS_8 samples are shown (cf. Figures S20–S25). There is no obvious difference in particle size or morphology as well as sample composition. All samples show a microstructure that is similar to *tetra*-Li_7SiPS_8 samples from the literature. It comprises inter-grown particles that are entrapped in some form of matrix that lies over them like a blanket. This would be consistent with the crystalline and amorphous phases determined by Rietveld refinement.[S19,S20]

In the SEM images and EDX maps (cf. Figures S20–S25), a few darker particles can be seen, suggesting the presence of a phase comprised of lighter atoms than the main phase. The particles can also be found in the EDX spectra, where it can be seen that those particles are elemental carbon. This carbon most likely stems from contamination of the SEM or the transport holder and is not an intrinsic part of the sample. In general, the EDX spectra of all samples look extremely similar, showing P to Si to S ratios of approximately 1:1:8. Only sample 27 shows areas with a slight shift in the P to Si ratio, however, averaged over the whole sample, the difference compared to the other samples is not significant. The homogeneity of the samples in the EDX and SEM images also highlights the similarity of the elemental composition of the phases determined by Rietveld refinement.

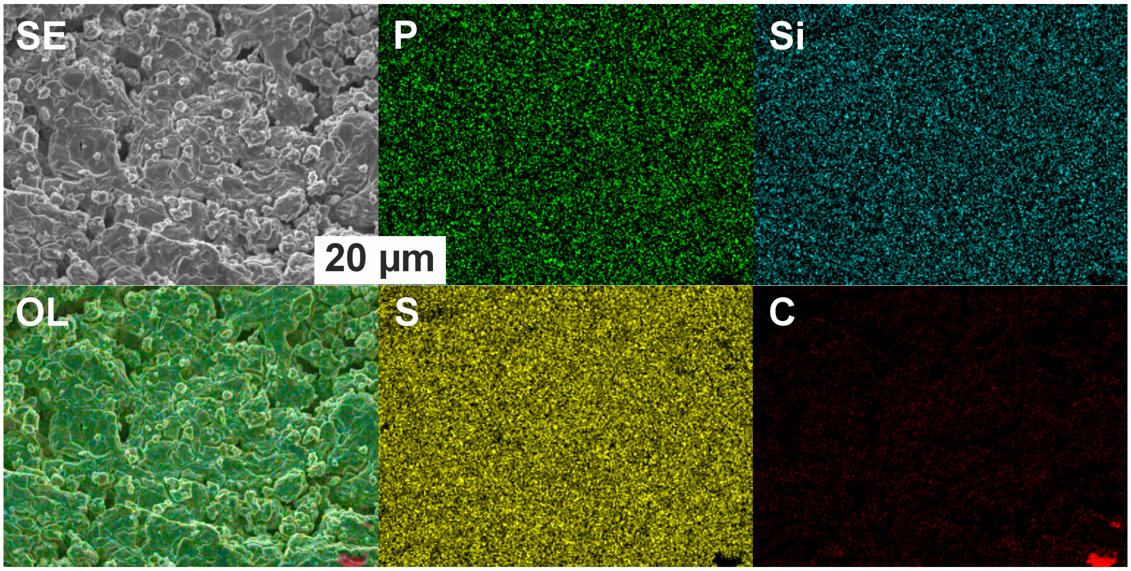

Figure S20: Secondary electron (SE) as well as an EDX overlay map (OL) and single element EDX maps of sample 6.

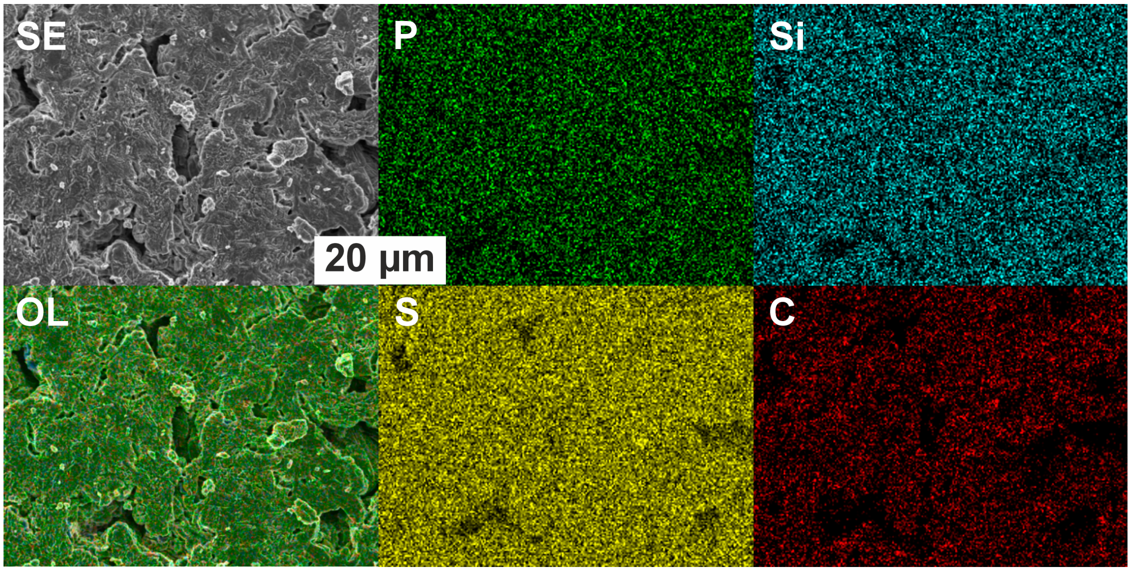

Figure S21: Secondary electron (SE) as well as an EDX overlay map (OL) and single element EDX maps of sample 18.

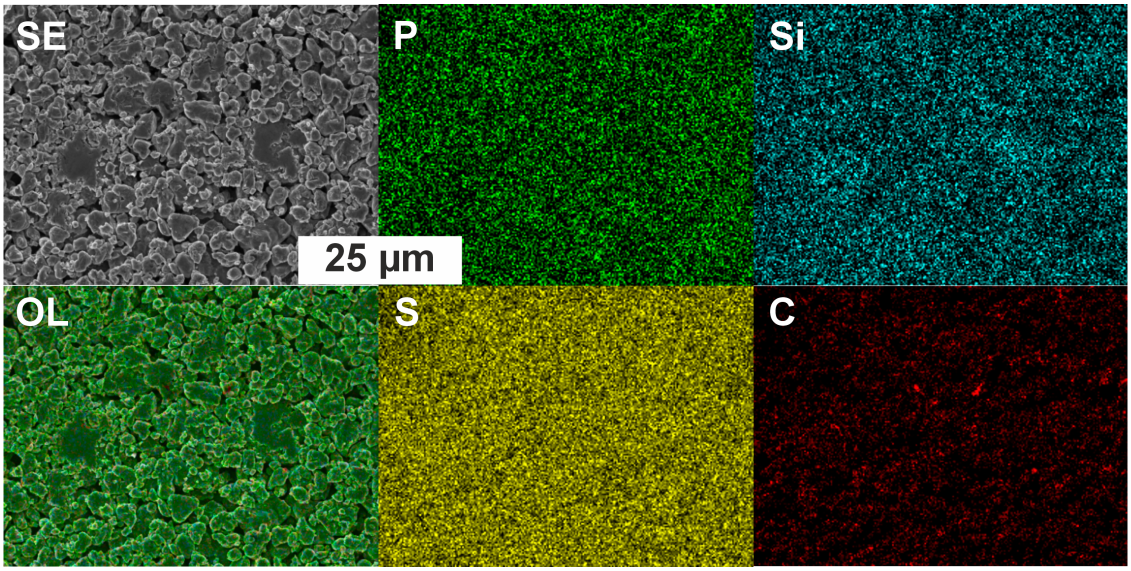

Figure S22: Secondary electron (SE) as well as an EDX overlay map (OL) and single element EDX maps of sample 27.

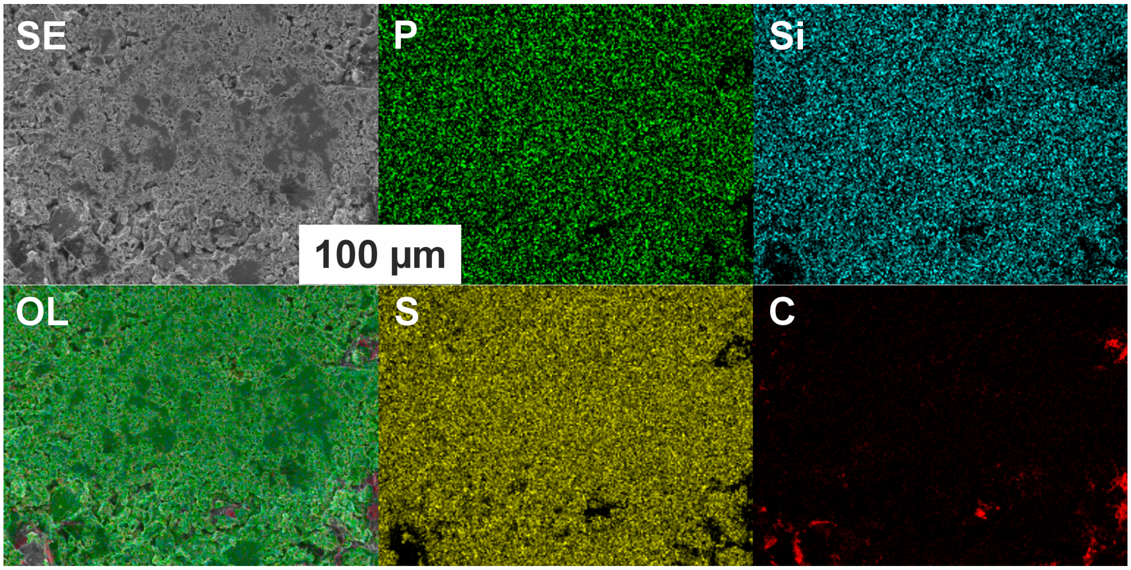

Figure S23: Secondary electron (SE) as well as an EDX overlay map (OL) and single element EDX maps of sample 30.

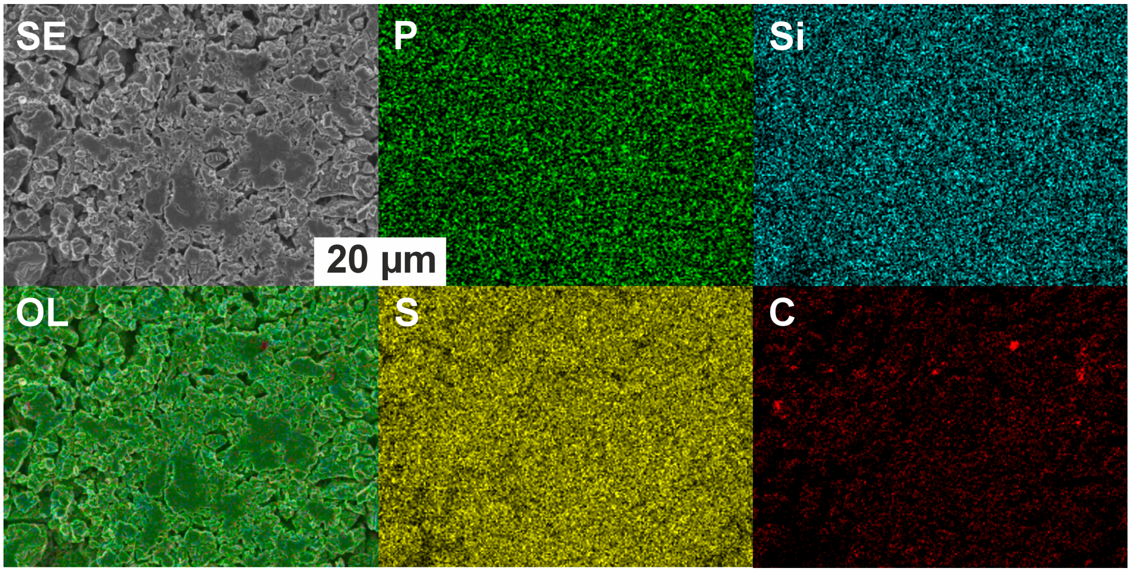

Figure S24: Secondary electron (SE) as well as an EDX overlay map (OL) and single element EDX maps of sample 31.

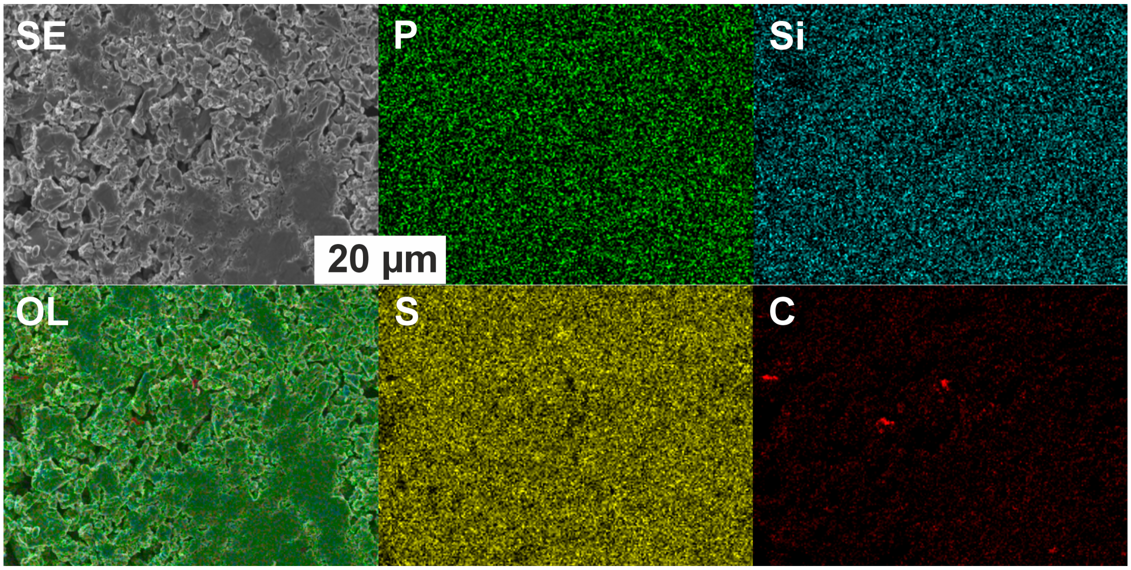

Figure S25: Secondary electron (SE) as well as an EDX overlay map (OL) and single element EDX maps of sample 32.

ssNMR

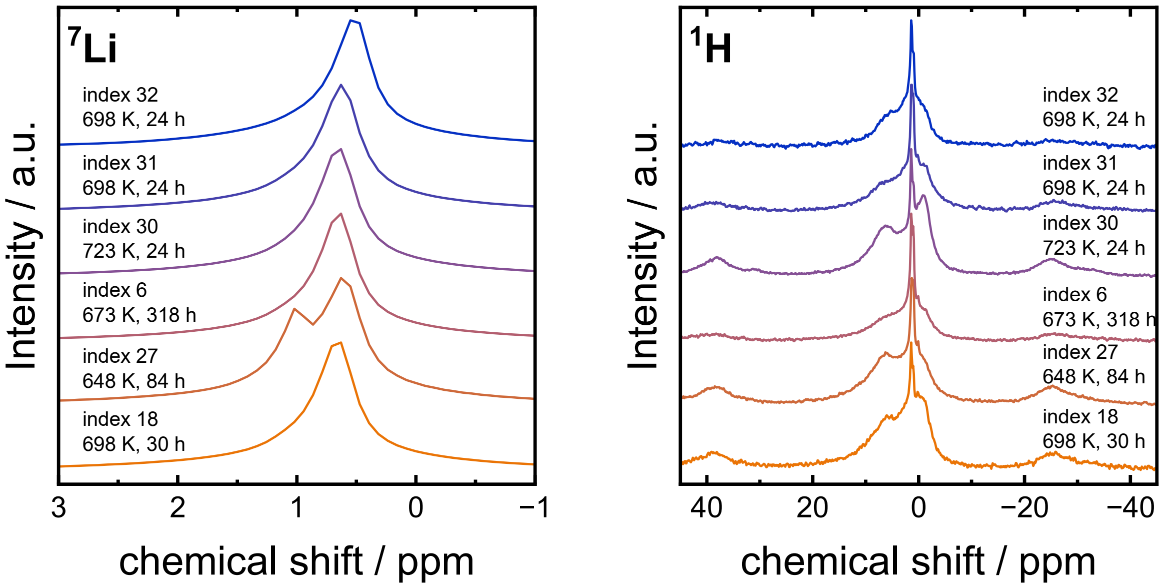

Figure S26: ^{7}Li and ^{1}H solid-state NMR spectra of the 6 samples that were analyzed in more detail.

Figure S26 shows the ^{7}Li NMR spectra of the samples. They exhibit characteristics very similar to those of the ^{6}Li NMR spectra (cf. Figure 5), but while they show higher signal intensity, they also have lower resolution and are therefore not discussed separately.

Figure S26 shows the ^{1}H NMR spectra of the samples. Despite working under dry and inert atmosphere, a certain amount of H atoms in the samples is nevertheless present as was previously also seen by Hatz et al..[S21] From this measurement the total amount of H atoms was derived and normalized by the measured sample weight to get values that can be easily compared (cf. Table S13). The values are in the same order of magnitude, indicating no significant difference in e.g. sample degradation that could explain the different ionic conductivities. It also rules out differences in water content as a reason for the different ionic conductivities as was described by Joos et al..[S22]. The signals might stem from adsorbed H_2O (very broad, ≈ 4.7 ppm[S23–S26]) and H_2S (≈ 1.2 ppm)[S27]. A small shoulder at ≈ 0.1 ppm is associated with hydroxide ions.[S26] An additional broad signal at around -1 ppm could not

be assigned. It is, however, difficult to reliably determine peak positions due to the weak signals and the background subtraction.

Table S13: Number of H atoms per mg derived from the ^{1}H NMR measurements.

index	H atoms/mg
6	2.23E+17
18	2.48E+17
27	4.37E+17
30	4.57E+17
31	2.44E+17
32	1.07E+17

Table S14: Comparison of amorphous phase content derived from ^{31}P MAS NMR and Rietveld refinement from XRPD data using the internal standard method. The approximate sum formula of the amorphous phase was assumed to be Li_3PS_4.

index	NMR [a%]	NMR [wt%]	Rietveld [wt%]
6	8.6	4.5	0
18*	32.2	19.0	27.0
27	8.1	4.2	2.8
30	6.3	3.2	7.7
31	9.2	4.7	−1.7
32	8.2	4.2	8.6
mean		4.2	3.5
SD		0.52	4.1

*Due to peak overlap in the ^{31}P MAS NMR, the values represents the combined amounts of *ortho*-Li_7SiPS_8 and the amorphous phase. The sample was omitted for the calculation of the mean value and the standard deviation (SD).

PFG NMR

Table S15: Isotropic ion diffusion radius and ionic conductivity ($H_R = 1$) calculated from the diffusion coefficient determined by PFG NMR.

sample index	r_{rms} at 303K [nm]	$\sigma_{NMR,303K}$ [$mScm^{-1}$]
6	1348.0	6.69
18	1285.69	6.08
27	722.08	1.92
30	1250.68	5.76
31	1298.46	6.21
32	1312.59	6.34

Rietveld refinement of synchrotron XRPD and PDF

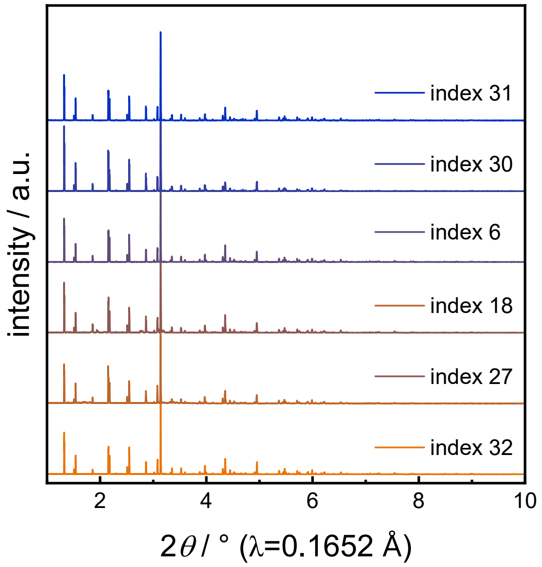

Figure S27: Synchrotron PXRD patterns of the studied subset in the 2θ range of 1.5 to 10°.

As discussed in the main text, new reflections in the synchrotron X-ray powder diffractograms (cf. Figures 3 and S27) become visible when the y-axis is changed from linear to logarithmic scaling. This is exemplarily shown below for one sample. The Rietveld refinements of all samples can be seen in Figures 4 and S28. The former figure exemplarily shows

why an LPS-like phase had to be used for refinement next to the t-Li_7SiPS_8 phase. While no additional signals are visible in a linear representation of the intensities, they become visible when the logarithm of the intensities is plotted. This is due to the stark anisotropic peak broadening and the small phase fractions.

The analysis of the pair distribution function (PDF) data can be seen in Figures S29–S34. For analysis, r values smaller than 0.5 Å were omitted due to the presence of termination ripples. The analysis nicely matches the structure of *tetra*-Li_7SiPS_8, especially for medium and high r values.

However, the difference curve of the PDF refinements shows significant differences up to 5 Å. This is probably caused by the presence of an amorphous phase that was already seen in ssNMR (cf. Figure 5) and quantitative Rietveld analysis of the laboratory XRPD (cf. Figures 2 and S1–S11). The chemical shift of the signal that was assigned to the amorphous phase in ^{31}P SSNMR implies the presence of PS_4 tetrahedra. Therefore, the PDF was refined again with fixed parameters for the *tetra*-Li_7SiPS_8 phase and an artificial structure in space group $P1$ with 2 independently rotating rigid bodies of PS_4 tetrahedra. To mimic the missing long-range order of the amorphous phase, the lattice parameters were refined and capped at 30 Å due to refinement performance. The structure was refined using simulated annealing. The procedure resulted in a much better fit of the low r region (cf. Figures S29–S34).

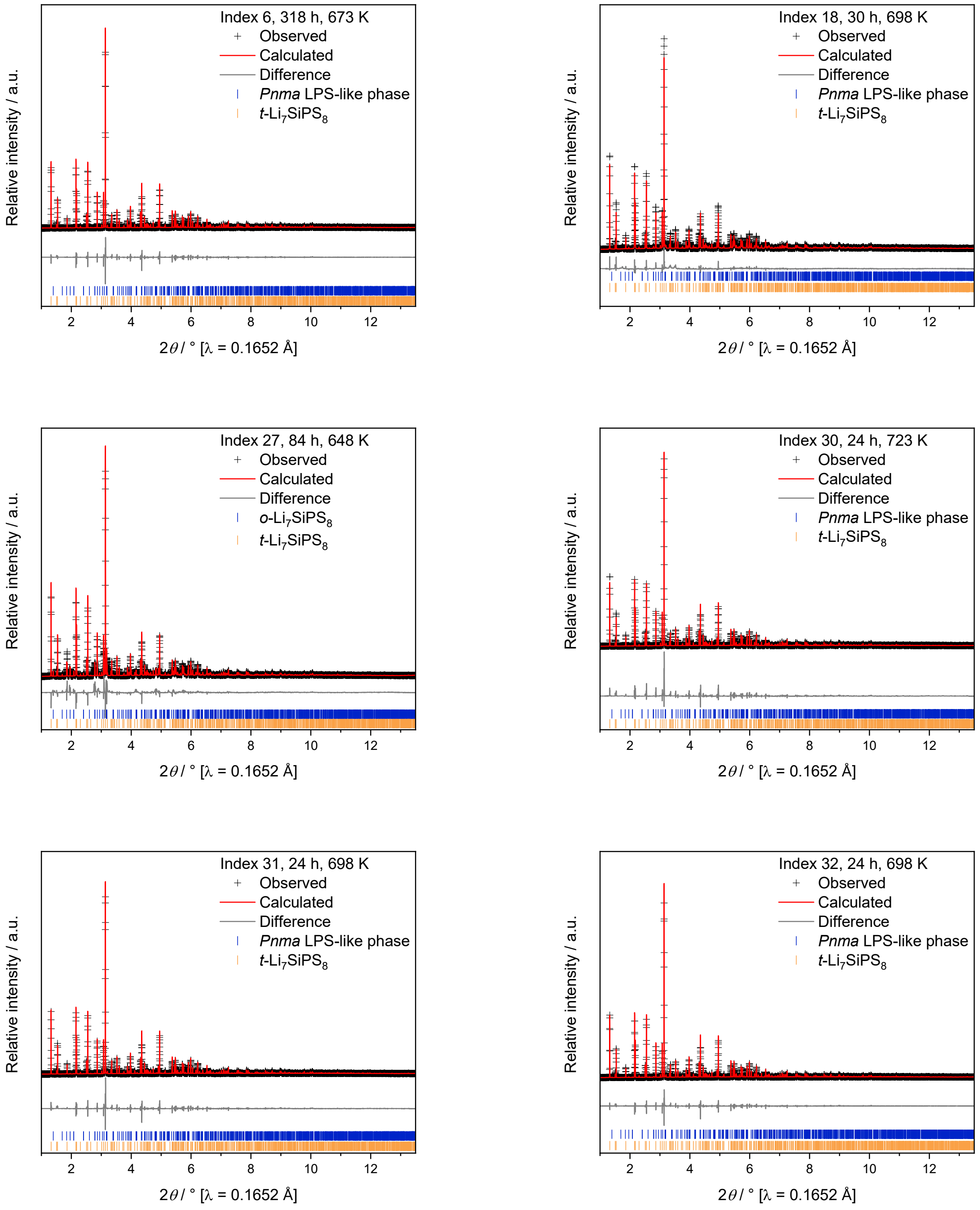

Figure S28: Rietveld refinement of the synchrotron PXRD data of the samples that were analyzed in more detail. The corresponding tables with information about the Rietveld refinements can be found in Tables S16–S17.

Table S16: Additional crystallographic information obtained from the quantitative Rietveld refinements of synchrotron PXRD data of samples 6 and 18, 27. The standard deviations are given in parentheses.

Samples index		6	18	27
Temperature program		318 h, 673 K	30 h, 698 K	84 h, 648 K
Phase / wt%	t-Li_7SiPS_8	98.86(8)	96.9(1)	88.1(3)
Space group		$P4_2/nmc$ (No. 132)		
Lattice parameters / Å	a	8.70041(4)	8.69802(9)	8.6981(1)
	c	12.5608(1)	12.5623(2)	12.5554(3)
Volume / $Å^3$		950.82(1)	950.41(3)	949.91(4)
R_{Bragg}		4.7189	6.3641	10.4222
Phase		LPS-like	LPS-like	o-Li_7SiPS_8
Phase / wt%		1.14(8)	3.1(1)	11.9(3)
Space group		$Pnma$ (No. 62)		
Lattice parameters / Å	a	13.57(1)	13.607(5)	13.5578(5)
	b	7.859(8)	7.840(1)	7.8661(3)
	c	6.109(5)	6.129(2)	6.1233(3)
Volume / $Å^3$		651(1)	653.7(4)	653.03(4)
R_{Bragg}		8.1394	8.3659	21.2309
Diffractometer		beamline ID31 at the ESRF, $\lambda = 0.1652$ Å		
Refined 2θ region / °		0.5-13.5		
R_p		13.050	8.465	15.488
R_{wp}		11.057	9.647	15.640
Number of refined parameters		29	31	28
Number of refined background parameters		8	8	8

Table S17: Additional crystallographic information obtained from the quantitative Rietveld refinements of synchrotron XRPD data of samples 30 and 31, 32. The standard deviations are given in parentheses.

Samples index		30	31	32
Temperature program		24 h, 723 K	24 h, 698 K	24 h, 698 K
Phase / wt%	t-Li_7SiPS_8	98.4(2)	97.8(1)	97.86(9)
Space group		$P4_2/nmc$ (No. 132)		
Lattice parameters / Å	a	8.69869(5)	8.69934(5)	8.69686(4)
	c	12.5603(1)	12.5612(1)	12.5586(1)
Volume / Å^3		950.40(2)	950.61(1)	949.87(1)
R_{Bragg}		12.9243	6.5356	4.9464
Phase / wt%	LPS-like	1.6(2)	2.2(1)	2.14(9)
Space group		$Pnma$ (No. 62)		
Lattice parameters / Å	a	13.569(7)	13.558(4)	13.550(3)
	b	7.89(1)	7.859(4)	7.852(3)
	c	6.107(8)	6.122(2)	6.126(1)
Volume / Å^3		653(1)	652.3(5)	651.7(3)
R_{Bragg}		12.9827	12.045	9.6055
Diffractometer	beamline ID31 at the ESRF, $\lambda = 0.1652$ Å			
Refined 2θ region / °		0.5-13.5		
R_p		16.053	14.209	12.290
R_{wp}		14.892	13.908	12.805
Number of refined parameters		29	29	29
Number of refined background parameters		8	8	8

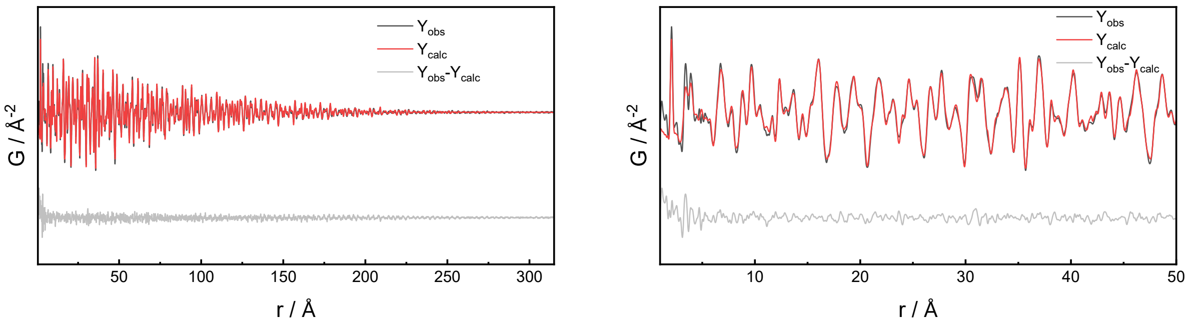

Figure S29: Refinement of the PDF data of the sample with index 6 over the entire range and a magnification for the range between 1 and 50 Å.

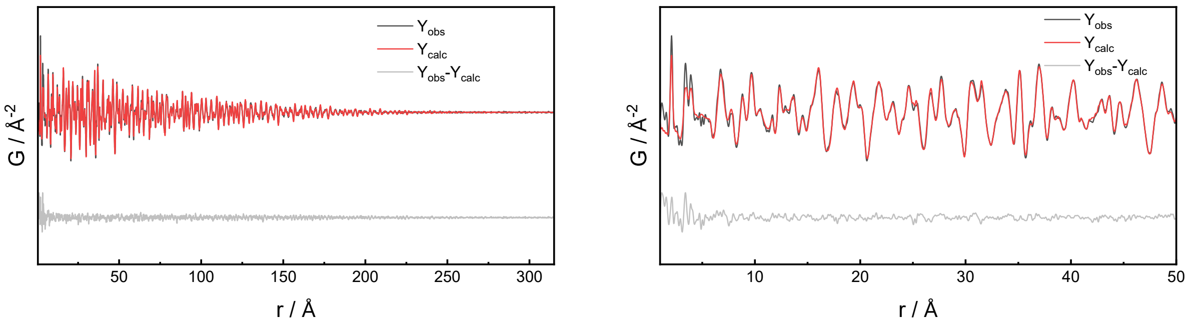

Figure S30: Refinement of the PDF data of the sample with index 18 over the entire range and a magnification for the range between 1 and 50 Å.

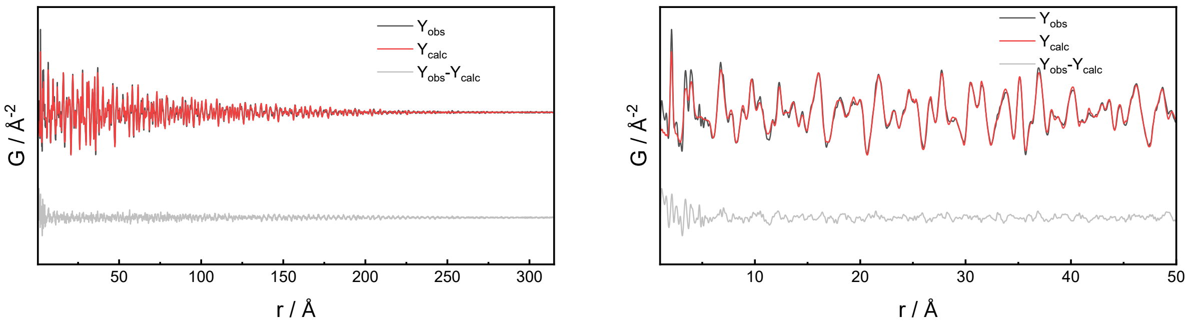

Figure S31: Refinement of the PDF data of the sample with index 27 over the entire range and a magnification for the range between 1 and 50 Å.

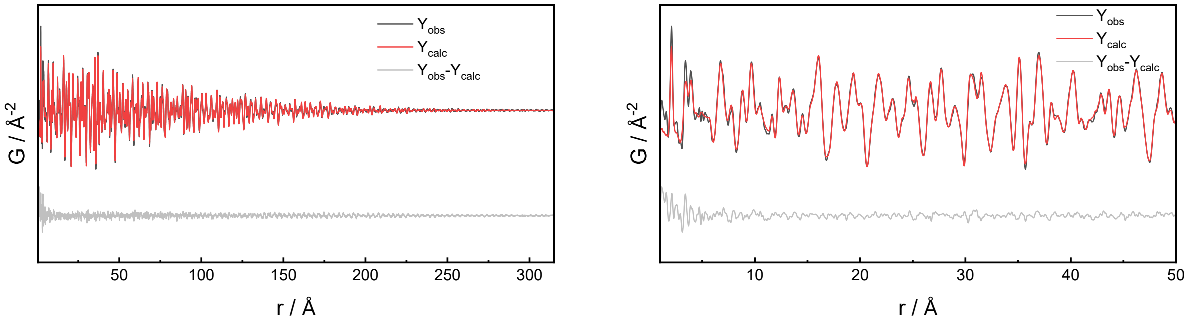

Figure S32: Refinement of the PDF data of the sample with index 30 over the entire range and a magnification for the range between 1 and 50 Å.

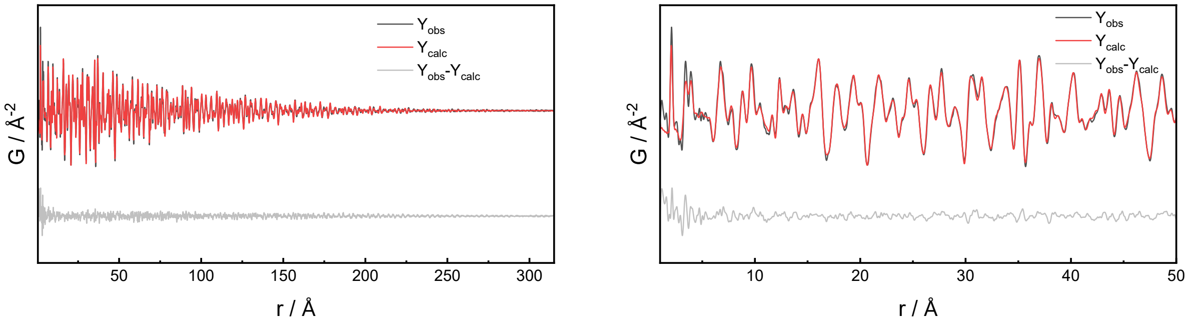

Figure S33: Refinement of the PDF data of the sample with index 31 over the entire range and a magnification for the range between 1 and 50 Å.

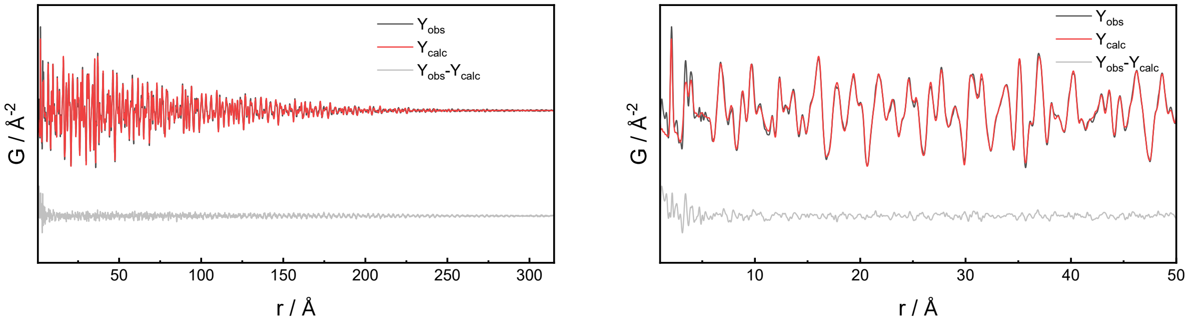

Figure S34: Refinement of the PDF data of the sample with index 32 over the entire range and a magnification for the range between 1 and 50 Å.

References

(S1) Brug, G.; van den Eeden, A.; Sluyters-Rehbach, M.; Sluyters, J. *Journal of Electroanalytical Chemistry and Interfacial Electrochemistry* **1984**, *176*, 275–295.

(S2) Irvine, J. T. S.; Sinclair, D. C.; West, A. R. *Advanced Materials* **1990**, *2*, 132–138.

(S3) Nuernberg, R. B. *Ionics* **2020**, *26*, 2405–2412.

(S4) Coelho, A. A. *Journal of Applied Crystallography* **2018**, *51*, 210–218.

(S5) Momentum Transfer GmbH. 2025; https://momentum-transfer.com/, https://momentum-transfer.com/, Accessed: Dec. 10, 2025.

(S6) Terban, M. W.; Billinge, S. J. L. *Chemical Reviews* **2022**, *122*, 1208–1272.

(S7) Egami, T.; Billinge, S. J. L. *Underneath the Bragg peaks*; Pergamon Materials; Pergamon, 2003.

(S8) Marlton, F.; Ivashko, O.; Zimmerman, M. v.; Gutowski, O.; Dippel, A.-C.; Jørgensen, M. R. V. *Journal of Applied Crystallography* **2019**, *52*, 1072–1076.

(S9) Peterson, P. F.; Božin, E. S.; Proffen, T.; Billinge, S. J. L. *Journal of Applied Crystallography* **2003**, *36*, 53–64.

(S10) Billinge, S. J. L.; Farrow, C. L. *Journal of Physics: Condensed Matter* **2013**, *25*, 454202.

(S11) Juhás, P.; Davis, T.; Farrow, C. L.; Billinge, S. J. L. *Journal of Applied Crystallography* **2013**, *46*, 560–566.

(S12) Lorch, E. *Journal of Physics* **1969**, *2*, 229–237.

(S13) Tanner, J. E. *The Journal of Chemical Physics* **1970**, *52*, 2523–2526.

(S14) Stejskal, E. O.; Tanner, J. E. *The Journal of Chemical Physics* **1965**, *42*, 288–292.

(S15) Shahriari, B.; Swersky, K.; Wang, Z.; Adams, R. P.; De Freitas, N. *Proceedings of the IEEE* **2016**, *104*, 148–175.

(S16) Rasmussen, C. E.; Williams, C. K. I. *Gaussian processes for machine learning*; MIT press Cambridge, MA, 2006; Vol. 1.

(S17) Jones, D. R.; Schonlau, M.; Welch, W. J. *Journal of Global Optimization* **1998**, *13*, 455–492.

(S18) Srinivas, N.; Krause, A.; Kakade, S. M.; Seeger, M. Gaussian process optimization in the bandit setting: No regret and experimental design. Proceedings of the 27th international conference on machine learning (ICML-10). 2010; pp 1015–1022.

(S19) Harm, S.; Hatz, A. K.; Moudrakovski, I.; Eger, R.; Kuhn, A.; Hoch, C.; Lotsch, B. V. *Chemistry of Materials* **2019**, *31*, 1280–1288.

(S20) Calaminus, R.; Harm, S.; Fabini, D. H.; Balzat, L. G.; Hatz, A.-K.; Duppel, V.; Moudrakovski, I.; Lotsch, B. V. *Chemistry of Materials* **2022**, *34*, 7666–7677.

(S21) Hatz, A.-K.; Calaminus, R.; Feijoo, J.; Treber, F.; Blahusch, J.; Lenz, T.; Reichel, M.; Karaghiosoff, K.; Vargas-Barbosa, N. M.; Lotsch, B. V. *ACS Applied Energy Materials* **2021**, *4*, 9932–9943.

(S22) Joos, M.; Kang, X.; Merkle, R.; Maier, J. *Nature Materials* **2025**, *24*, 821–834.

(S23) Hoffman, R. E. *Magnetic Resonance in Chemistry* **2006**, *44*, 606–616.

(S24) Xu, M.; Harris, K. D.; Thomas, J. M. *Solid State Nuclear Magnetic Resonance* **2009**, *35*, 93–99.

(S25) Yoder, C. H.; Pasteris, J. D.; Worcester, K. N.; Schermerhorn, D. V. *Calcified Tissue International* **2011**, *90*, 60–67.

(S26) Wilson, E. E.; Awonusi, A.; Morris, M. D.; Kohn, D. H.; Tecklenburg, M. M.; Beck, L. W. *Biophysical Journal* **2006**, *90*, 3722–3731.

(S27) Hahn, J. *Zeitschrift für Naturforschung B* **1985**, *40*, 263–272.
